# Supplementary material for: A resilient battery electric bus transit system configuration
Source: Nat Commun. 2023 Dec 13;14:8279. doi: 10.1038/s41467-023-43924-6 (PMC10719261; doi:10.1038/s41467-023-43924-6)
Supplement: Supplementary file 2 — Supplementary Information [file 41467_2023_43924_MOESM2_ESM.pdf]

A Resilient Battery Electric Bus Transit System Configuration

Supplementary Discussion 1: Charging system disruption

There are several reasons that could lead to the partial failure of BEB's charging system while the remaining charging network is fully functional. These reasons could be summarized into two categories: utility grid disruption and charging component disruption.

First, the disruption of the utility grid could affect a part of the BEB charging system (specific area) for various reasons ranging from technical issues to external factors. Some of these reasons and real-world examples are as follows:

Equipment failure of the power grid distribution infrastructure, such as transformers, substations, and circuit breakers, could affect a specific region in the city. For example, an equipment failure in the Hydro Ottawa Richmond Road substation on March 7, 2020, in Ottawa, Canada, affected nearly 12,000 customers for around 20 hours in 5 regions [1]. Scheduled maintenance or repairs to the utility grid infrastructure could require power shutting down to a specific area. For example, the BC Hydro Power Smart, British Columbia, Canada, provides a plan for the number of scheduled electricity outages, region, duration, and number of customers affected [2]. Scheduled load shedding is sometimes used to manage overload and prevent a widespread blackout. This involves shutting down the power to specific regions for a certain period to balance the load. For example, in Chengdu, China, due to extremely high temperatures, 100 electric vehicle charging poles were out of service or limited power during August 2022. Similarly, in Egypt, during the summer of 2023, the government mandated a load-shedding program due to the severe heatwave and significant increase in electricity consumption. The following schedule determines power outage regions for each hour.

Many weather-related factors, such as heavy winds, lightning strikes, ice accumulation, or flooding, can damage power lines and equipment, resulting in specific area outages. For example, in Toronto, Canada, parts of the downtown area were left without power due to strong winds in Southern Ontario in December 2022 [3]. Other factors, such as power line theft, animal interference, human error, and equipment malfunction (faulty relay or sensor), could lead to a specific area of electricity outage. A comprehensive data on major power outage events in the continental U.S. is presented in [4]. In general, power outages are rising due to climate change, increased energy demand, and an aging electrical grid. Do et. al., 2023 analyzed USA outage data for 2,447 counties from 2018-2020. The results show 17,484 8+ hour outages, most of which co-occur with weather-related issues (62.1%) [5].

Second, scheduled maintenance is required for various components, which could lead to a partial charging service disruption. For example, according to the ABB E-Bus Charger User and Operation Manual for EU products, the charger must be inspected and serviced yearly, including cabinet inspection, components testing, and emergency stop inspection. Moreover, the charger must be checked before reuse in some cases, such as lighting struck, damage due to accident or fire, and its location flooding.

The work of Raman et. al, 2022 highlights the effect of flooding on public electric vehicle charging stations. The authors emphasize that flooding could make chargers unavailable (out of service due to water damage) or disrupt access to the charging stations [6].

Supplementary Table 1

Supplementary Table 1. Sample of previous studies on BEB system configuration with uncertainty

Author	System configuration	Infrastructure allocation	Charging/Fleet Scheduling	Uncertain parameter	Uncertainty distribution or set	Optimization model
Zhou [7]	Number of chargers<br>Power of chargers		Charging & Fleet scheduling	Travel time<br>Battery degradation	Gaussian distribution	Two-stage stochastic programming
Gairola and Nezamuddin [8]	Battery capacity<br>Power of chargers<br>Number of chargers	✓	Charging scheduling	Energy consumption	Budgeted set	Robust model
Liu [9]			Charging scheduling	Energy consumption	Budgeted set	Robust model
Zhou [10]	Number of chargers<br>Battery capacity		Charging scheduling	Energy consumption	Box set<br>Budgeted set<br>Ellipsoidal set	Robust model
Hu [11]	Battery capacity	✓	Charging scheduling	Boarding & alighting time.<br>Travel time	Budgeted sets	Robust model
Bai [12]	Battery capacity<br>Number of buses	✓<br>Dynamic Wireless Charging	Charging & Fleet scheduling	Energy consumption<br>Power supply fluctuation	Budgeted sets	Robust model
Kong [13]	Charge and discharge power	✓	Charging scheduling	Energy consumption	Gaussian distribution	Chance-constrained programming
Zheng [14]			Charging scheduling	Charging time	Uniform distribution<br>Gaussian distribution	Stochastic linear programming
Iliopoulou and Kepaptsoglou [15]		✓	Charging & Fleet scheduling	Power supply fluctuation	Budgeted set	Robust model
An [16]	Number of Buses	✓	Charging scheduling	Energy consumption	Uniform distribution	Stochastic integer programming
Liu [17]	Battery capacity	✓	Charging scheduling	Energy consumption	Budgeted set	Robust model
Liu and Song [18]	Battery capacity	✓<br>DWCI	Charging scheduling	Energy consumption<br>Charging time	Box sets<br>Budgeted sets	Robust model
This study	Number of chargers<br>Power of chargers<br>Battery capacity	✓	Charging scheduling	Charging station disruption	Budgeted set	Two-stage robust model

Supplementary Discussion 2: Case Study

The proposed robust BEB system configuration model is applied to a real-world, large-scale bus transit network in Oakville City, Ontario, Canada. The transit network data, including the operational timetable and distances, are extracted from the REMIX platform based on Oakville's General Transit Feed Specification (GTFS) data. The network operates 38 bus routes served by 91 buses and 1,240 bus stops. The number of buses per route varies from one to six, and the route length varies from 6.38 km to 53.64 km, as presented in Supplementary Table 2. The total number of daily trips and vehicle kilometers travelled (VKT) on a weekday are 1,349 trips and 15,694 km, respectively.

Supplementary Table 2. Oakville Transit network data

Route ID	Route Name	Route length (km)	Vehicle Count (#)	Route ID	Route Name	Route length (km)	Vehicle Count (#)
1	Trafalgar	11.794	2	26	Falgarwood	10.535	2
2	Lakeshore	53.638	4	28	Glen Abbey North	21.502	2
3	Third Line	19.154	4	32	Burloak – Great Lakes	27.885	2
4	Speers-Cornwall	33.311	4	33	Palermo	17.047	1
5	Dundas	19.790	1	71	White Oaks S. Special	13.548	1
6	Upper Middle	40.089	4	80E	Holy Trinity East	22.905	1
10	West Industrial	20.583	3	80W	Holy Trinity West	21.901	1
11	Linbrook	21.127	4	81A	Abbey Park and Loyola 81A	10.102	1
13	Westoak Trails	34.011	5	81B	Abbey Park and Loyola 81B	8.491	1
14	Lakeshore West	15.438	5	81N	Abbey Park North 81N	16.222	1
15	Bridge	25.601	4	81S	Abbey Park / Loyola South	8.433	1
17	Kerr	7.817	2	82	Loyola North	14.136	1
18	Glen Abbey South	18.736	2	83	Blakelock West	6.381	2
19	River Oaks	26.229	6	84	O. T. H. S.	12.929	1
20	Northridge	20.461	4	86	Garth Webb	23.932	1
21	Clearview	21.269	2	91	Oakville Senior's Special	27.138	1
22	Upper Glen Abbey	17.925	2	102	Winston Park	18.506	2
24	South Common	33.866	5	120	East Industrial	16.405	2
25	Aspen Forest	21.942	2	190	River Oaks Express	20.568	2

As mentioned in the Methods, Subsection Base model, selecting the locations of candidate charging stations is based on three measures. First, bus stops, terminals, or hubs with a higher weighted degree of centrality are chosen. The weighted degree centrality of each station in the Oakville network (1,240 locations) is calculated, and stations with a weighted degree higher than 20 are selected (21 locations). In addition, for each route, the start, end, and any other en-route stops with lengthy recovering times were included in the candidate charging station set. This was done while guaranteeing that the count of candidate locations of each route should be higher than $k+1$. In the case study, the maximum k equals 2. Therefore, each route should include at least three candidate locations of charging stations. Toward this end, a total of 77 en-route locations are identified as candidate charging locations.

Due to the lack of real-world BEB energy consumption data for the Oakville transit, the energy consumption rate parameters e^{base} and e^{batt} are estimated using a linear regression model for data extracted from a BEB simulator. Advanced Vehicle Simulator (ADVISOR) MATLAB/Simulink environment is utilized to extract the BEB energy consumption data for various battery capacities. ADVISOR was first developed by the National Renewable Energy Laboratory (NREL) and used in the literature for simulating the BEB conduct [19, 20]. The Orange County Cycle 1-Hz speed profile is used in the simulation process [21].

The temporal values of the WTT GHG emission (ρ_t^{em}) are estimated based on the hourly distribution of the electricity generation sources obtained from the Ontario Power Generation (OPG) public data with 65 \$ per tCO_2e 2023 Carbon Pollution Price. While the electricity ToU tariff (ρ_t^{elect}) is based on the Ontario winter weekday electricity fees. The values of the sets A^{batt} and A^{st} are assumed to include a wide range of values representing the entire market without any restriction to a specific manufacturer. The remaining input parameters used in the proposed model are presented in Supplementary Table 3.

The parameter δ (number of workdays) is taken as 365. However, this does not mean the charging station failure will last for the entire year. This value is used in the solution process of the proposed two-stage robust model by multiplying the daily operational cost of the worst-case failure scenario by 365 to provide the worst-case annual operational costs. However, as mentioned in the Methods, Subsection Two-stage robust model, the actual annual operational costs of the resilient model are estimated after the realization of the charging station failure scenario each day.

Supplementary Table 3. Input values of model parameters

Parameters	Value	Reference	Parameters	Value	Reference
ρ^{st}	\$75,000	[22]	ϑ^{max}	90%	[23]
ρ^{ch}	300 \$ per kW	[24]	ϑ^{min}	20%	[23]
ρ^{po}	\$13,000	[24]	τ	0.106	[24]
ρ^{batt}	500\$ per kWh	[25]	λ_1	3	[9]
ρ^{bus}	550,000\$ per bus	[25]	T_s	$\frac{2}{60}$ hour	Model Specific
η^{ch}	95%	[9]	δ	365	Model Specific
P_{po}^{max}	500	Model Specific	N_i^{max}	10	Model Specific
ρ^{pen}	\$1,000,000	Model Specific	A^{batt}	{100,200,…,700}	Model Specific
Q^{max}	700	Model Specific	A^{st}	{250,500,…,1500}	Model Specific

Moreover, the failed bus penalty cost (ρ^{pen}) is set to 1 million dollars (a suitably large value) to ensure a full operation is satisfied. This is achieved by preventing the NC&CG algorithm from convergence until guaranteeing that there are no failed buses under any charging station failure scenario.

Supplementary Discussion 3: Solution algorithm convergence

The designed nested column-and-constraint generation (NC&CG) algorithm is utilized to solve the proposed two-stage RO model to obtain a resilient BEB system configuration. Two Robust Models are solved under two different levels of conservativeness ($k = 1$ and $k = 2$) of the charging station failure uncertainty set. The algorithm convergence for both models is illustrated in Supplementary Fig. 1.

The NC&CG solution algorithm decomposes the two-stage RO model into two problems: the master problem (MP) solved in the outer-level C&CG algorithm that provides the lower bound (LB) of the model, and the sub-problem (SP) handled by the inner-level C&CG algorithm and provides the upper bound (UB) of the model. The algorithm gradually reduces the difference between the UB and the LB iteratively until it reaches a small value, at which point it terminates. Specifically, in Supplementary Fig. 1A, the Robust model with $k = 1$ converges in six iterations after adding only 11 charging station failure scenarios to the MP. Similarly, the Robust Model with $k = 2$ terminates in iteration 13 after adding 27 scenarios of two simultaneous charging station failures to the MP, as visualized in Supplementary Fig. 1B. These results emphasize that the NC&CG algorithm converges to the optimal solution in a finite number of iterations without the need to add all the feasible scenarios of the uncertainty set.

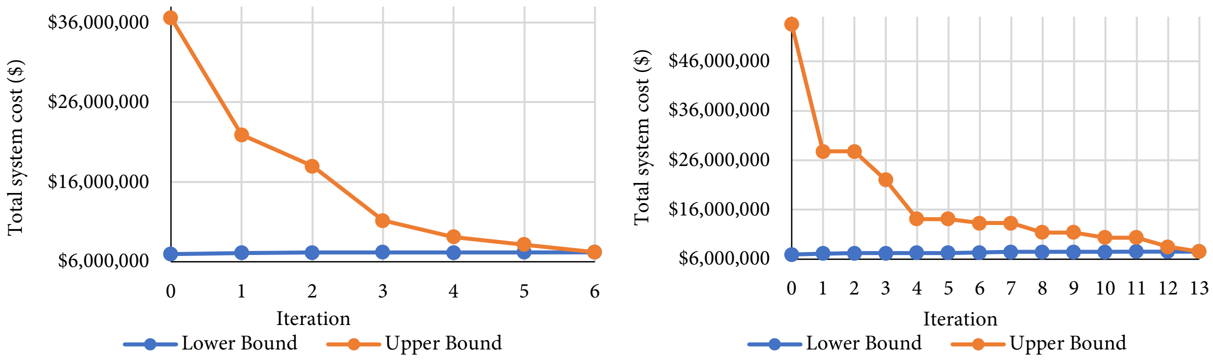

(a) $k = 1$ (b) $k = 2$

Supplementary Fig. 1 Solution algorithm convergence (Robust Models)

Legend: During the solution algorithm, iteratively, the upper bound reduces and the lower bound increases until the termination criterion is met (the relative optimality gap is lower than a predefined small ratio). a) The Robust Model with $k = 1$ (one station failure) converges in six iterations. b) The Robust Model with $k = 2$ (two stations failure) converges in 13 iterations.

In Supplementary Fig. 1, it is obvious that the upper bound (UB) reduces significantly from the initial iteration to the final one. In comparison, the lower bound (LB) increases at a relatively lower rate. For example, in the Robust Model with $k = 1$, the difference between the initial iteration and final iteration of the LB and UB are $227,207 and $29,409,346, respectively. This phenomenon is attributed to the nature of the UB and LB problems.

Specifically, the objective function of the master problem (MP) in each iteration provides the LB. The MP is designed to obtain the BEB system configuration in a pre-perturbation form to handle the failure scenarios, and the final iteration solution is the resilient model design that is robust against any k charging station disruption. Therefore, the objective function increases slowly through the algorithm iterations while handling more added failure scenarios. In other words, in the MP, all the BEB system configuration variables are utilized as decision variables, including the first-stage variables (allocated charging stations, charger-rated power in each station, number of charger poles, and the BEB fleet battery capacities) and the second-stage variables (charging schedule and failed buses). Therefore, the MP has the entire flexibility of decision variables to optimize the total system costs under the included set of charging station failure scenarios in each iteration. Therefore, as a pre-perturbation design, the total system costs of the MP (LB) increase slowly by adding more failure scenarios, and the relative ratio between the last iteration objective function (Robust Model) and the initial one (Base Model) is the price of robustness (PoR). Most notably, the BEB system configuration obtained by the MP in each iteration does not include any failed buses (high penalty cost).

On the other hand, the objective function of the sub-problem (SP) in each iteration provides the UB. The SP is designed to obtain the worst-case failure scenario under the resulting BEB system design from the MP. This failure scenario will be added to the MP in the next iteration. Therefore, the SP is formulated in a maximization of the post-perturbation effect way. In other words, iteratively, the SP takes the first-stage variables (allocated charging stations, charger-rated power in each station, number of charger poles, and the BEB fleet battery capacities) from the MP as parameters and estimates the worst-case charging station failure scenario using the second-stage variables (the failure random variable, charging

schedule, and failed buses). With the high penalty cost of the failed buses and the SP's maximization nature, the SP's objective function (UB) is relatively higher than the MP (LB), especially in the first iterations. After that, the MP updates the first stage variables iteratively, making the BEB system design more robust. Therefore, the SP objective function decreases dramatically until the final iteration with a fully resilient model when the objective function of the MP (pre-perturbation survivable design) is approximately equal to the SP (maximum post-perturbation effect).

Supplementary Figure 2

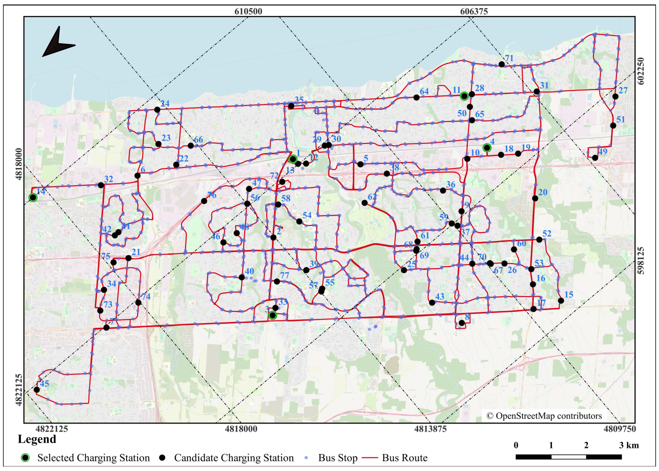

a) Base Model

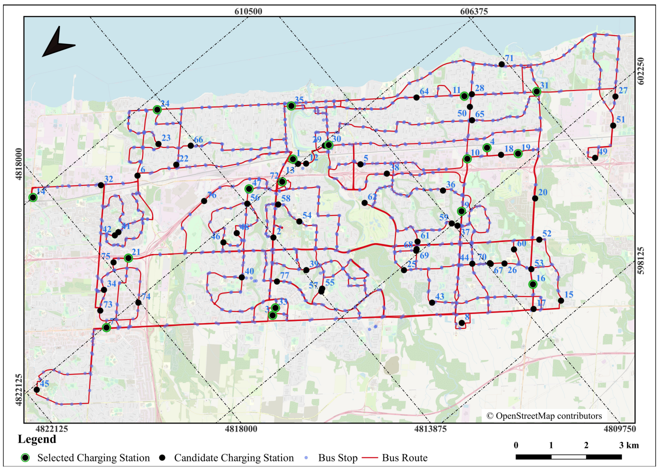

b) Robust Model $k = 1$

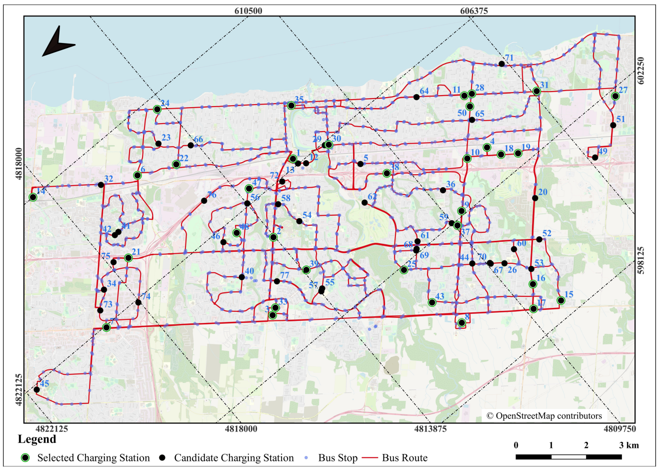

c) Robust Model $k = 2$

Supplementary Fig. 2 Charging stations spatial distribution

Legend: Each map illustrates the transit network: routes as red lines, bus stops as small blue dots, while the locations of the candidate charging stations are marked in black dots numbered from 1 to 77, and the selected stations are displayed as black dots surrounded by green circles. a) Represents the selected charging stations of the Base Model with nominal operation. b) Represents the selected charging stations of the Robust Model with one charging station failure budget ($k = 1$). c) Represents the selected charging stations of the Robust Model with two simultaneous charging station failure budget ($k = 2$). The maps are drawn using QGIS [26] used under the GNU General Public License Version 2 [27]. The GTFS data for Oakville City used to draw the transit network elements is publicly available [28] and used under the Town of Oakville Open Data Licence [29]. The OpenStreetMap is used in the maps under the Open Data Commons Open Database License (ODBL) by the OpenStreetMap Foundations (OSMF) [30].

Supplementary Table 4

Supplementary Table 4. Charging station configuration and utilization (Base Model)

Station ID	Station Name	Charger unit power (kW)	Number of poles (#)	Number of charging events (#/day)	Total charging duration (min/day)	Number of buses charging (#)	Daily energy demand (kWh)
1	Oakville GO Station	500	2	318	1,458	50	4,837.015
3	Uptown Core Terminal	500	1	153	570	17	3,242.350
4	Bronte GO Station	500	3	179	1,440	27	5,679.782
11	South Oakville Centre	500	1	172	654	16	3,168.622
14	Clarkson GO Station	250	1	52	218	8	694.438

Supplementary Table 5

Supplementary Table 5. Charging station configuration and utilization (Robust Model, $k = 1$) – Nominal operation

Station ID	Station Name	Charger unit power (kW)	Number of poles (#)	Number of charging events (#/day)	Total charging duration (min/day)	Number of buses charging (#)	Daily energy demand (kWh)
1	Oakville GO Station	500	2	215	740	47	2,807.447
3	Uptown Core Terminal	500	1	147	422	17	1,542.180
4	Bronte GO Station	500	1	142	538	28	2,555.978
7	Dundas St (east) at Hampshire Gate	500	1	84	266	12	889.162
9	3rd Line at Greenridge Cr	250	1	143	296	10	581.136
10	3rd Line at Wyecroft Rd	250	1	103	206	15	390.244
11	South Oakville Centre	250	1	147	488	15	1,426.091
14	Clarkson GO Station	250	1	40	94	8	176.742
16	Bronte Rd at Khalsa Gate	500	1	29	112	3	380.962
19	Wyecroft Rd at Pacific Rd	500	1	120	298	17	953.632
21	Upper Middle Rd (east) at Buckingham	250	1	75	150	8	290.460
24	Lakeshore Rd (east) at Charnwood	250	1	83	302	10	657.494
30	Speers Rd at Kerr St	250	1	149	366	16	868.155
31	Rebecca St at Bronte Rd	500	1	99	210	7	748.125
33	Taunton Rd at Hays Blvd	500	1	184	418	17	1,128.755
35	Church St at Dunn St	500	1	61	180	8	665.286
47	Iroquois Shore Rd at 8th Line	250	1	103	226	6	538.482
72	Oakville Place	250	1	68	270	6	611.960

Supplementary Table 6

Supplementary Table 6. Charging station configuration and utilization (Robust Model, $k = 2$) – Nominal operation

Station ID	Station Name	Charger unit power (kW)	Number of poles (#)	Number of charging events (#/day)	Total charging duration (min/day)	Number of buses charging (#)	Daily energy demand (kWh)
1	Oakville GO Station	500	1	201	538	48	1,805.250
2	Trafalgar Rd at White Oaks Blvd	500	1	183	454	6	791.893
3	Uptown Core Terminal	500	1	139	360	17	906.793
4	Bronte GO Station	250	1	134	448	28	1,120.944
6	Ford Dr at Royal Windsor Dr	500	1	126	264	13	861.100
7	Dundas St (east) at Hampshire Gate	500	1	84	264	12	723.070
8	Oakville Trafalgar Memorial Hospital	250	1	44	170	4	255.605
9	3rd Line at Greenridge Cr	250	1	143	296	10	506.000
10	3rd Line at Wyecroft Rd	250	1	103	206	15	305.590
11	South Oakville Centre	250	1	134	390	15	1,018.372
14	Clarkson GO Station	250	1	46	144	9	241.925
15	Colonel William at Stalybridge Dr	250	1	26	72	4	116.667
16	Bronte Rd at Khalsa Gate	250	1	25	86	3	206.057
17	Dundas St (west) at Bronte Rd	250	1	34	112	4	232.130
18	Wyecroft Rd at Westgate Rd	250	1	74	148	12	253.750
19	Wyecroft Rd at Pacific Rd	500	1	119	298	17	1,040.575
21	Upper Middle Rd at Buckingham Rd	500	1	75	150	8	545.748
22	Maple Grove Village	250	1	34	156	8	226.896
24	Lakeshore Rd (east) at Charnwood	500	1	82	302	10	669.347
25	Westoak Trails Blvd at Treetop Terrace	250	1	72	276	7	480.096
27	Burloak Ave at Fothergill Blvd	250	1	42	146	5	313.978
28	Rebecca St at 3rd Line	250	1	52	104	7	136.569
30	Speers Rd at Kerr St	500	1	154	370	17	807.315
31	Rebecca St at Bronte Rd	250	1	101	210	7	453.538
33	Taunton Rd at Hays Blvd	500	1	190	418	17	663.376
35	Church St at Dunn St	500	1	68	180	8	358.653
37	Glen Abbey Gate at 3rd Line	250	1	41	88	4	85.075
38	North Service Rd (west) at 4th Line	500	1	86	250	7	307.415
39	River Oaks Blvd (east) at Pelee Blvd	250	1	76	218	8	351.806
43	Pine Glen Rd at Proudfoot Trail	250	1	20	76	4	65.087
47	Iroquois Shore Rd at 8th Line	250	1	105	226	6	269.904
48	Lancaster Dr at Fairbanks Pl	250	1	35	70	3	58.481
50	3rd Line at Tansley Dr	250	1	96	192	6	258.521

Supplementary Table 7

Supplementary Table 7. Number of charging events (Robust Model, $k = 1$) – No disruption

Hour	Station ID																
	1	3	4	7	9	10	11	14	16	19	21	24	30	31	33	35	47	72
05:00	1	2	1	0	0	0	2	0	0	1	1	0	0	1	1	0	0	0
06:00	23	10	13	3	10	7	12	2	1	6	3	8	8	5	8	5	7	5
07:00	19	11	15	7	14	10	9	4	2	13	7	8	13	7	13	5	8	4
08:00	4	7	7	7	14	11	7	4	2	10	6	7	9	6	11	4	8	3
09:00	2	6	4	7	6	6	7	2	1	8	3	6	8	6	11	4	6	4
10:00	0	5	3	4	6	4	6	1	2	6	3	3	9	6	12	4	6	5
11:00	15	11	10	5	6	5	10	2	1	6	3	2	9	6	10	2	6	4
12:00	18	9	7	3	6	4	9	1	2	5	3	4	8	6	12	2	6	4
13:00	12	8	7	5	6	5	9	2	1	6	3	4	8	6	10	2	6	4
14:00	16	9	7	3	6	4	10	1	2	5	3	4	8	6	12	3	6	4
14:00	15	11	7	5	9	5	9	2	2	5	4	4	9	7	11	2	6	4
15:00	17	9	10	6	11	6	7	5	2	8	8	5	12	6	11	2	6	4
16:00	0	11	8	8	16	8	7	3	1	9	8	11	12	7	13	4	6	6
17:00	3	10	9	6	12	7	8	2	2	10	7	9	10	7	11	6	10	5
18:00	18	8	11	5	9	8	7	2	1	9	3	4	8	5	12	5	5	4
19:00	13	5	5	2	4	4	6	2	2	4	3	2	6	4	7	4	3	2
20:00	12	5	7	4	3	4	7	2	2	4	3	2	5	3	6	3	3	2
21:00	13	5	6	2	3	3	8	1	2	3	3	0	6	3	6	2	3	2
22:00	9	5	5	2	2	2	6	2	1	2	1	0	1	2	6	1	2	1
23:00	5	0	0	0	0	0	1	0	0	0	0	0	0	0	1	1	0	1

Supplementary Table 8

Supplementary Table 8. Number of charging events (Robust Model, $k = 1$) – Under disruption of Station ID 1

Hour	Station ID																
	3	4	7	9	10	11	14	16	19	21	24	30	31	33	35	47	72
05:00	2	1	0	0	0	2	0	0	1	1	0	0	1	1	0	0	0
06:00	10	13	3	10	7	12	2	1	6	3	8	8	5	8	5	7	5
07:00	10	14	7	14	10	12	4	2	13	7	8	13	7	12	5	9	4
08:00	6	9	7	14	11	8	4	2	10	6	7	9	6	11	4	8	3
09:00	7	4	7	6	6	7	2	1	9	3	6	7	6	11	4	6	4
10:00	5	3	4	6	4	6	1	2	6	3	3	9	6	12	4	6	5
11:00	9	11	5	6	5	10	2	1	6	3	2	9	6	10	2	5	4
12:00	9	6	3	6	4	9	1	2	5	3	4	9	6	12	2	6	4
13:00	8	8	5	6	5	9	2	1	6	3	4	9	6	10	2	5	4
14:00	9	8	3	6	4	10	1	2	5	3	4	9	6	12	3	5	4
14:00	10	9	5	9	5	9	2	2	5	4	4	9	7	11	2	5	4
15:00	10	11	6	11	6	9	5	2	8	8	5	13	6	11	2	5	4
16:00	11	8	8	16	8	7	3	1	9	8	11	12	7	13	4	6	6
17:00	9	9	6	12	7	9	2	2	10	7	9	10	7	11	6	10	5
18:00	8	13	5	9	8	7	2	1	9	3	4	8	5	11	5	5	4
19:00	5	6	2	4	4	7	2	2	4	3	2	6	5	7	4	3	2
20:00	5	8	4	3	4	7	2	2	4	3	2	5	4	6	3	3	2
21:00	5	6	2	3	3	8	1	2	3	3	0	6	4	7	2	3	2
22:00	5	5	2	2	2	6	2	1	2	1	0	1	2	7	1	2	1
23:00	0	0	0	0	0	1	0	0	0	0	0	0	0	1	1	0	1

Supplementary Table 9

Supplementary Table 9. Energy demand distribution of Bus ID 3 under disruption (Robust Model, $k = 1$)

Disruption Scenarios	Charging demand per station			
	4	7	19	24
No disruption	163.79 kWh	33.33 kWh	118.52 kWh	97.18 kWh
Disruption to Station ID 4	NA	50.00 kWh	234.61 kWh	128.22 kWh
Disruption to Station ID 7	173.92 kWh	NA	125.06 kWh	113.85 kWh
Disruption to Station ID 19	276.59 kWh	16.67 kWh	NA	119.57 kWh
Disruption to Station ID 24	178.54 kWh	55.74 kWh	178.54 kWh	NA

Supplementary Table 10

Supplementary Table 10. Energy demand distribution of Bus ID 3 under disruption (Robust Model, $k = 2$)

Disruption Scenarios	Charging demand per station				
	4	6	7	19	24
No disruption	66.67 kWh	40.49 kWh	20.13 kWh	180.31 kWh	105.22 kWh
Disruption to Stations ID 4 & 6	NA	NA	33.33 kWh	187.72 kWh	191.77 kWh
Disruption to Stations ID 4 & 7	NA	91.07 kWh	NA	185.24 kWh	136.52 kWh
Disruption to Stations ID 4 & 19	NA	144.32 kWh	45.88 kWh	NA	222.62 kWh
Disruption to Stations ID 4 & 24	NA	138.43 kWh	66.67 kWh	207.72 kWh	NA
Disruption to Stations ID 6 & 7	97.72 kWh	NA	NA	139.61 kWh	175.50 kWh
Disruption to Stations ID 6 & 19	179.60 kWh	NA	33.90 kWh	NA	199.32 kWh
Disruption to Stations ID 6 & 24	196.48 kWh	NA	58.94 kWh	157.40 kWh	NA
Disruption to Stations ID 7 & 19	156.42 kWh	116.18 kWh	NA	NA	140.23 kWh
Disruption to Stations ID 7 & 24	125.00 kWh	125.23 kWh	NA	162.59 kWh	NA
Disruption to Stations ID 19 & 24	229.26 kWh	127.84 kWh	55.72 kWh	NA	NA

Supplementary Figure 3

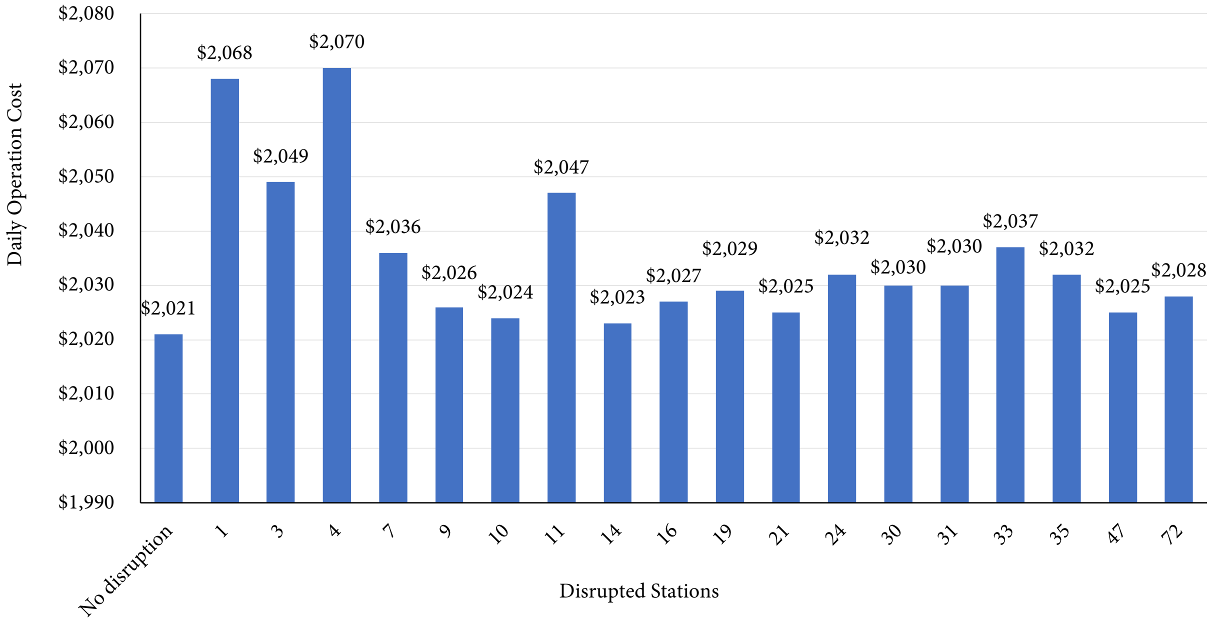

Supplementary Fig. 3 Daily operation costs under any disruption scenario (Robust Model, $k = 1$)

Legend: The daily operational costs are related to charging station disruption scenarios. In the x-axis, all the alternative scenarios of one charging station failure are shown, including the No disruption scenario. The y-axis illustrates the daily operational costs of each disruption scenario (presented as a bar). The lower total daily operational costs is $2,021 in the No disruption scenario, while the maximum operational cost is $ 2,070 associated with charging station ID 4 disruption scenario.

Supplementary Table 11

Supplementary Table 11. Daily operation cost under all disruption scenarios (Robust Model, $k = 2$)

Disrupted Stations	Daily operational cost	Disrupted Stations	Daily operational cost	Disrupted Stations	Daily operational cost	Disrupted Stations	Daily operational cost	Disrupted Stations	Daily operational cost	Disrupted Stations	Daily operational cost	Disrupted Stations	Daily operational cost
No disrupt.	$1,881	(3, 19)	$1,894	(7, 9)	$1,891	(9, 50)	$1,885	(15, 24)	$1,887	(19, 25)	$1,893	(27, 33)	$1,885
(1, 2)	$1,901	(3, 21)	$1,894	(7, 10)	$1,890	(10, 11)	$1,889	(15, 25)	$1,886	(19, 27)	$1,888	(27, 35)	$1,887
(1, 3)	$1,910	(3, 22)	$1,889	(7, 11)	$1,894	(10, 14)	$1,886	(15, 27)	$1,883	(19, 28)	$1,887	(27, 37)	$1,883
(1, 4)	$1,917	(3, 24)	$1,894	(7, 14)	$1,891	(10, 15)	$1,882	(15, 28)	$1,881	(19, 30)	$1,891	(27, 38)	$1,884
(1, 6)	$1,909	(3, 25)	$1,893	(7, 15)	$1,888	(10, 16)	$1,884	(15, 30)	$1,885	(19, 31)	$1,890	(27, 39)	$1,885
(1, 7)	$1,906	(3, 27)	$1,889	(7, 16)	$1,889	(10, 17)	$1,884	(15, 31)	$1,884	(19, 33)	$1,889	(27, 43)	$1,882
(1, 8)	$1,899	(3, 28)	$1,887	(7, 17)	$1,888	(10, 18)	$1,885	(15, 33)	$1,884	(19, 35)	$1,888	(27, 47)	$1,884
(1, 9)	$1,900	(3, 30)	$1,891	(7, 18)	$1,890	(10, 19)	$1,889	(15, 35)	$1,882	(19, 37)	$1,887	(27, 48)	$1,883
(1, 10)	$1,900	(3, 31)	$1,890	(7, 19)	$1,896	(10, 21)	$1,889	(15, 37)	$1,881	(19, 38)	$1,888	(27, 50)	$1,884
(1, 11)	$1,910	(3, 33)	$1,897	(7, 21)	$1,899	(10, 22)	$1,884	(15, 38)	$1,883	(19, 39)	$1,889	(28, 30)	$1,887
(1, 14)	$1,901	(3, 35)	$1,890	(7, 22)	$1,889	(10, 24)	$1,890	(15, 39)	$1,884	(19, 43)	$1,887	(28, 31)	$1,885
(1, 15)	$1,897	(3, 37)	$1,888	(7, 24)	$1,896	(10, 25)	$1,888	(15, 43)	$1,882	(19, 47)	$1,889	(28, 33)	$1,884
(1, 16)	$1,898	(3, 38)	$1,889	(7, 25)	$1,893	(10, 27)	$1,884	(15, 47)	$1,884	(19, 48)	$1,886	(28, 35)	$1,883
(1, 17)	$1,897	(3, 39)	$1,895	(7, 27)	$1,889	(10, 28)	$1,883	(15, 48)	$1,882	(19, 50)	$1,888	(28, 37)	$1,882
(1, 18)	$1,899	(3, 43)	$1,888	(7, 28)	$1,888	(10, 30)	$1,887	(15, 50)	$1,882	(21, 22)	$1,888	(28, 38)	$1,883
(1, 19)	$1,904	(3, 47)	$1,890	(7, 30)	$1,892	(10, 31)	$1,886	(16, 17)	$1,883	(21, 24)	$1,894	(28, 39)	$1,884
(1, 21)	$1,904	(3, 48)	$1,888	(7, 31)	$1,891	(10, 33)	$1,886	(16, 18)	$1,885	(21, 25)	$1,892	(28, 43)	$1,882
(1, 22)	$1,899	(3, 50)	$1,889	(7, 33)	$1,891	(10, 35)	$1,884	(16, 19)	$1,888	(21, 27)	$1,889	(28, 47)	$1,884
(1, 24)	$1,904	(4, 6)	$1,901	(7, 35)	$1,889	(10, 37)	$1,883	(16, 21)	$1,889	(21, 28)	$1,887	(28, 48)	$1,881
(1, 25)	$1,906	(4, 7)	$1,907	(7, 37)	$1,888	(10, 38)	$1,884	(16, 22)	$1,884	(21, 30)	$1,890	(28, 50)	$1,882
(1, 27)	$1,899	(4, 8)	$1,893	(7, 38)	$1,889	(10, 39)	$1,885	(16, 24)	$1,889	(21, 31)	$1,890	(30, 31)	$1,892
(1, 28)	$1,897	(4, 9)	$1,897	(7, 39)	$1,890	(10, 43)	$1,883	(16, 25)	$1,888	(21, 33)	$1,890	(30, 33)	$1,887
(1, 30)	$1,911	(4, 10)	$1,896	(7, 43)	$1,888	(10, 47)	$1,885	(16, 27)	$1,884	(21, 35)	$1,888	(30, 35)	$1,890
(1, 31)	$1,901	(4, 11)	$1,903	(7, 47)	$1,889	(10, 48)	$1,883	(16, 28)	$1,883	(21, 37)	$1,887	(30, 37)	$1,885
(1, 33)	$1,901	(4, 14)	$1,895	(7, 48)	$1,888	(10, 50)	$1,884	(16, 30)	$1,885	(21, 38)	$1,888	(30, 38)	$1,887
(1, 35)	$1,903	(4, 15)	$1,892	(7, 50)	$1,889	(11, 14)	$1,890	(16, 31)	$1,885	(21, 39)	$1,889	(30, 39)	$1,886
(1, 37)	$1,897	(4, 16)	$1,893	(8, 9)	$1,886	(11, 15)	$1,888	(16, 33)	$1,885	(21, 43)	$1,887	(30, 43)	$1,885
(1, 38)	$1,900	(4, 17)	$1,893	(8, 10)	$1,884	(11, 16)	$1,889	(16, 35)	$1,884	(21, 47)	$1,888	(30, 47)	$1,885
(1, 39)	$1,903	(4, 18)	$1,900	(8, 11)	$1,890	(11, 17)	$1,887	(16, 37)	$1,882	(21, 48)	$1,887	(30, 48)	$1,885
(1, 43)	$1,897	(4, 19)	$1,920	(8, 14)	$1,886	(11, 18)	$1,890	(16, 38)	$1,884	(21, 50)	$1,887	(30, 50)	$1,886
(1, 47)	$1,901	(4, 21)	$1,909	(8, 15)	$1,883	(11, 19)	$1,895	(16, 39)	$1,885	(22, 24)	$1,891	(31, 33)	$1,887
(1, 48)	$1,897	(4, 22)	$1,893	(8, 16)	$1,884	(11, 21)	$1,894	(16, 43)	$1,883	(22, 25)	$1,887	(31, 35)	$1,885
(1, 50)	$1,898	(4, 24)	$1,904	(8, 17)	$1,883	(11, 22)	$1,888	(16, 47)	$1,884	(22, 27)	$1,884	(31, 37)	$1,884
(2, 3)	$1,894	(4, 25)	$1,905	(8, 18)	$1,884	(11, 24)	$1,894	(16, 48)	$1,883	(22, 28)	$1,882	(31, 38)	$1,885
(2, 4)	$1,895	(4, 27)	$1,893	(8, 19)	$1,888	(11, 25)	$1,893	(16, 50)	$1,883	(22, 30)	$1,886	(31, 39)	$1,886
(2, 6)	$1,889	(4, 28)	$1,893	(8, 21)	$1,888	(11, 27)	$1,894	(17, 18)	$1,884	(22, 31)	$1,885	(31, 43)	$1,885
(2, 7)	$1,894	(4, 30)	$1,898	(8, 22)	$1,883	(11, 28)	$1,889	(17, 19)	$1,888	(22, 33)	$1,885	(31, 47)	$1,886
(2, 8)	$1,885	(4, 31)	$1,900	(8, 24)	$1,888	(11, 30)	$1,896	(17, 21)	$1,887	(22, 35)	$1,884	(31, 48)	$1,884
(2, 9)	$1,887	(4, 33)	$1,894	(8, 25)	$1,887	(11, 31)	$1,893	(17, 22)	$1,883	(22, 37)	$1,883	(31, 50)	$1,885
(2, 10)	$1,885	(4, 35)	$1,894	(8, 27)	$1,884	(11, 33)	$1,890	(17, 24)	$1,888	(22, 38)	$1,884	(33, 35)	$1,885
(2, 11)	$1,890	(4, 37)	$1,891	(8, 28)	$1,882	(11, 35)	$1,908	(17, 25)	$1,886	(22, 39)	$1,885	(33, 37)	$1,884
(2, 14)	$1,887	(4, 38)	$1,893	(8, 30)	$1,885	(11, 37)	$1,888	(17, 27)	$1,883	(22, 43)	$1,883	(33, 38)	$1,886
(2, 15)	$1,883	(4, 39)	$1,894	(8, 31)	$1,885	(11, 38)	$1,888	(17, 28)	$1,881	(22, 47)	$1,884	(33, 39)	$1,889
(2, 16)	$1,885	(4, 43)	$1,892	(8, 33)	$1,885	(11, 39)	$1,891	(17, 30)	$1,884	(22, 48)	$1,883	(33, 43)	$1,884
(2, 17)	$1,883	(4, 47)	$1,894	(8, 35)	$1,884	(11, 43)	$1,888	(17, 31)	$1,884	(22, 50)	$1,883	(33, 47)	$1,886
(2, 18)	$1,886	(4, 48)	$1,892	(8, 37)	$1,882	(11, 47)	$1,890	(17, 33)	$1,884	(24, 25)	$1,892	(33, 48)	$1,884
(2, 19)	$1,888	(4, 50)	$1,893	(8, 38)	$1,884	(11, 48)	$1,888	(17, 35)	$1,883	(24, 27)	$1,889	(33, 50)	$1,885
(2, 21)	$1,889	(6, 7)	$1,896	(8, 39)	$1,885	(11, 50)	$1,890	(17, 37)	$1,881	(24, 28)	$1,888	(35, 37)	$1,883
(2, 22)	$1,884	(6, 8)	$1,889	(8, 43)	$1,882	(14, 15)	$1,885	(17, 38)	$1,882	(24, 30)	$1,890	(35, 38)	$1,884
(2, 24)	$1,890	(6, 9)	$1,890	(8, 47)	$1,884	(14, 16)	$1,886	(17, 39)	$1,884	(24, 31)	$1,890	(35, 39)	$1,885
(2, 25)	$1,889	(6, 10)	$1,889	(8, 48)	$1,883	(14, 17)	$1,884	(17, 43)	$1,882	(24, 33)	$1,890	(35, 43)	$1,883
(2, 27)	$1,885	(6, 11)	$1,893	(8, 50)	$1,883	(14, 18)	$1,885	(17, 47)	$1,883	(24, 35)	$1,888	(35, 47)	$1,884
(2, 28)	$1,884	(6, 14)	$1,893	(9, 10)	$1,887	(14, 19)	$1,890	(17, 48)	$1,882	(24, 37)	$1,887	(35, 48)	$1,883
(2, 30)	$1,887	(6, 15)	$1,887	(9, 11)	$1,892	(14, 21)	$1,890	(17, 50)	$1,883	(24, 38)	$1,888	(35, 50)	$1,884
(2, 31)	$1,886	(6, 16)	$1,888	(9, 14)	$1,887	(14, 22)	$1,889	(18, 19)	$1,890	(24, 39)	$1,890	(37, 38)	$1,883
(2, 33)	$1,894	(6, 17)	$1,887	(9, 15)	$1,884	(14, 24)	$1,891	(18, 21)	$1,890	(24, 43)	$1,888	(37, 39)	$1,884
(2, 35)	$1,884	(6, 18)	$1,889	(9, 16)	$1,885	(14, 25)	$1,889	(18, 22)	$1,884	(24, 47)	$1,889	(37, 43)	$1,881
(2, 37)	$1,883	(6, 19)	$1,893	(9, 17)	$1,884	(14, 27)	$1,886	(18, 24)	$1,888	(24, 48)	$1,887	(37, 47)	$1,884
(2, 38)	$1,884	(6, 21)	$1,893	(9, 18)	$1,886	(14, 28)	$1,884	(18, 25)	$1,888	(24, 50)	$1,888	(37, 48)	$1,882
(2, 39)	$1,886	(6, 22)	$1,891	(9, 19)	$1,890	(14, 30)	$1,888	(18, 27)	$1,885	(25, 27)	$1,888	(37, 50)	$1,882
(2, 43)	$1,884	(6, 24)	$1,898	(9, 21)	$1,890	(14, 31)	$1,886	(18, 28)	$1,883	(25, 28)	$1,886	(38, 39)	$1,885
(2, 47)	$1,885	(6, 25)	$1,892	(9, 22)	$1,885	(14, 33)	$1,887	(18, 30)	$1,887	(25, 30)	$1,890	(38, 43)	$1,883
(2, 48)	$1,884	(6, 27)	$1,888	(9, 24)	$1,891	(14, 35)	$1,886	(18, 31)	$1,886	(25, 31)	$1,889	(38, 47)	$1,885
(2, 50)	$1,884	(6, 28)	$1,886	(9, 25)	$1,889	(14, 37)	$1,884	(18, 33)	$1,885	(25, 33)	$1,890	(38, 48)	$1,882
(3, 4)	$1,899	(6, 30)	$1,891	(9, 27)	$1,886	(14, 38)	$1,885	(18, 35)	$1,885	(25, 35)	$1,888	(38, 50)	$1,883
(3, 6)	$1,894	(6, 31)	$1,890	(9, 28)	$1,884	(14, 39)	$1,886	(18, 37)	$1,883	(25, 37)	$1,886	(39, 43)	$1,884
(3, 7)	$1,895	(6, 33)	$1,890	(9, 30)	$1,887	(14, 43)	$1,884	(18, 38)	$1,885	(25, 38)	$1,887	(39, 47)	$1,885
(3, 8)	$1,888	(6, 35)	$1,888	(9, 31)	$1,886	(14, 47)	$1,886	(18, 39)	$1,885	(25, 39)	$1,889	(39, 48)	$1,883
(3, 9)	$1,891	(6, 37)	$1,887	(9, 33)	$1,887	(14, 48)	$1,885	(18, 43)	$1,884	(25, 43)	$1,887	(39, 50)	$1,885
(3, 10)	$1,890	(6, 38)	$1,888	(9, 35)	$1,886	(14, 50)	$1,885	(18, 47)	$1,885	(25, 47)	$1,888	(43, 47)	$1,883
(3, 11)	$1,895	(6, 39)	$1,889	(9, 37)	$1,884	(15, 16)	$1,884	(18, 48)	$1,883	(25, 48)	$1,886	(43, 48)	$1,882
(3, 14)	$1,890	(6, 43)	$1,888	(9, 38)	$1,886	(15, 17)	$1,882	(18, 50)	$1,884	(25, 50)	$1,887	(43, 50)	$1,882
(3, 15)	$1,888	(6, 47)	$1,889	(9, 39)	$1,886	(15, 18)	$1,883	(19, 21)	$1,895	(27, 28)	$1,883	(47, 48)	$1,884
(3, 16)	$1,891	(6, 48)	$1,887	(9, 43)	$1,885	(15, 19)	$1,887	(19, 22)	$1,888	(27, 30)	$1,886	(47, 50)	$1,884
(3, 17)	$1,888	(6, 50)	$1,887	(9, 47)	$1,886	(15, 21)	$1,887	(19, 24)	$1,893	(27, 31)	$1,885	(48, 50)	$1,882
(3, 18)	$1,890	(7, 8)	$1,889	(9, 48)	$1,884	(15, 22)	$1,883						

Supplementary Table 12

Supplementary Table 12. Hourly energy demand in kWh (Base Model)

Hour	Charging Station ID					Total (kWh)
	1	3	4	11	14	
05:00	5	17	6	24	0	52
06:00	258	134	189	185	52	819
07:00	340	163	427	201	52	1183
08:00	340	233	423	217	52	1265
09:00	224	270	427	158	52	1131
10:00	140	106	230	105	33	614
11:00	340	270	410	227	17	1263
12:00	340	270	427	229	52	1319
13:00	340	270	427	229	50	1316
14:00	340	270	427	229	42	1308
14:00	287	270	427	229	50	1264
15:00	340	270	407	229	52	1299
16:00	183	39	115	55	45	437
17:00	246	54	265	95	52	712
18:00	273	225	377	192	33	1101
19:00	260	90	235	154	17	756
20:00	224	100	238	147	17	726
21:00	213	103	160	155	8	640
22:00	123	87	64	89	17	379
23:00	22	0	0	17	0	38

Supplementary Table 13

Supplementary Table 13. Hourly energy demand in kWh (Robust Model ($k = 1$) nominal operation)

Hour	Station ID																		Total (kWh)
	1	3	4	7	9	10	11	14	16	19	21	24	30	31	33	35	47	72	
05:00	5	15	6	0	0	0	15	0	0	2	8	0	0	3	0	0	0	0	55
06:00	232	85	146	53	49	33	113	17	19	85	25	67	75	43	71	62	42	60	1277
07:00	175	106	133	73	50	33	70	17	17	95	25	38	65	58	77	55	50	53	1188
08:00	49	40	17	56	41	16	27	1	12	5	8	53	18	50	67	10	42	0	512
09:00	0	0	0	1	0	8	17	0	0	0	0	0	2	46	27	1	0	0	101
10:00	0	0	0	0	0	0	0	0	0	0	0	0	0	0	0	0	0	0	0
11:00	273	156	287	73	50	33	126	17	33	95	25	33	79	58	102	67	50	60	1618
12:00	273	156	287	73	50	33	126	8	33	95	25	67	79	58	102	67	50	60	1643
13:00	273	156	287	73	50	33	126	17	33	95	25	67	79	58	102	67	50	60	1651
14:00	273	156	287	73	50	33	126	8	33	95	25	67	79	58	102	67	50	60	1643
14:00	273	156	287	73	50	33	126	17	33	95	25	67	79	58	102	67	50	60	1651
15:00	273	156	287	73	50	33	126	17	33	95	25	67	79	58	102	67	50	60	1651
16:00	0	0	0	0	0	0	0	0	0	0	0	0	0	0	0	0	0	0	0
17:00	10	8	0	8	0	8	8	0	0	4	0	17	10	15	1	4	0	9	101
18:00	178	103	209	73	50	31	84	17	0	67	22	58	66	50	83	40	34	48	1212
19:00	140	55	85	37	25	16	79	8	33	60	17	33	58	48	55	30	19	25	824
20:00	126	70	88	73	25	25	84	17	33	35	17	25	47	32	45	21	19	25	808
21:00	145	77	90	21	25	15	102	8	33	19	17	0	43	36	48	33	18	19	748
22:00	97	46	62	53	15	8	62	9	33	10	2	0	8	20	26	1	17	7	477
23:00	10	0	0	0	0	0	8	0	0	0	0	0	0	0	15	9	0	8	50

Supplementary Table 14

Supplementary Table 14. Hourly energy demand kWh (Robust Model ($k = 2$) nominal operation)

Hour	Station ID																																Total (kWh)
	1	2	3	4	6	7	8	9	10	11	14	15	16	17	18	19	21	22	24	25	27	28	30	31	33	35	37	38	39	43	47	48	50	
05:00	6	0	15	6	0	0	12	0	0	15	0	0	0	0	0	2	14	22	0	17	8	0	0	4	0	0	7	0	0	0	0	0	0	128
06:00	173	64	77	98	81	53	26	50	27	89	25	8	10	8	25	107	48	29	67	50	25	8	82	42	50	34	8	33	26	10	31	8	21	1494
07:00	98	65	31	34	49	58	23	39	13	41	17	8	15	17	12	94	36	0	13	7	8	0	20	28	50	0	0	0	25	10	0	0	19	833
08:00	10	41	20	0	7	22	17	3	10	0	0	8	9	17	6	0	0	0	48	0	0	0	0	0	30	0	0	0	0	8	0	0	8	263
09:00	0	24	0	0	0	0	0	0	0	0	0	8	4	17	0	0	0	0	0	0	0	0	0	0	0	0	0	0	0	0	0	0	0	54
10:00	0	0	0	0	0	0	0	0	0	0	0	0	0	0	0	0	0	0	0	0	0	0	0	0	0	0	0	0	0	0	0	0	0	0
11:00	173	65	80	117	81	65	26	50	30	90	17	8	15	17	25	108	50	0	67	50	25	13	82	42	56	34	8	33	33	0	31	8	22	1522
12:00	173	65	80	117	81	65	26	50	30	90	25	8	15	17	25	108	50	29	67	50	25	13	82	42	56	34	8	33	33	0	31	8	22	1559
13:00	173	65	80	117	81	65	26	50	30	90	25	8	15	17	25	108	50	29	67	50	25	13	82	42	56	34	8	33	33	0	31	8	22	1559
14:00	173	65	80	117	81	65	26	50	30	90	25	8	15	17	25	108	50	29	67	50	25	13	82	42	56	34	8	33	33	0	31	8	22	1559
14:00	173	65	80	117	81	65	26	50	30	90	25	8	15	17	25	108	50	29	67	50	25	13	82	42	56	34	8	33	33	10	31	8	22	1569
15:00	173	65	80	117	81	65	26	50	30	90	25	8	15	17	25	108	50	29	67	50	25	13	82	42	56	34	8	33	33	10	31	8	22	1569
16:00	0	0	0	0	0	0	0	0	0	0	0	0	0	0	0	0	0	0	0	0	0	0	0	0	0	0	0	0	0	0	0	0	0	0
17:00	8	0	18	8	0	0	0	0	0	0	0	0	8	3	0	0	0	0	12	0	1	0	0	4	0	0	0	0	5	0	0	0	0	69
18:00	173	38	77	90	65	60	12	48	14	63	25	8	10	8	25	88	40	15	55	47	25	11	64	25	50	34	8	16	22	8	8	0	18	1251
19:00	103	26	51	50	68	24	6	26	9	62	8	8	15	17	10	37	33	17	42	13	24	11	53	33	46	29	6	12	17	10	0	0	7	873
20:00	72	41	52	56	49	41	6	14	25	61	8	8	13	17	9	43	33	0	33	18	24	9	43	28	26	21	0	24	17	0	11	0	21	827
21:00	76	38	49	56	23	34	0	11	22	88	8	8	15	17	9	10	24	0	0	15	25	11	49	28	34	27	6	12	25	0	17	0	16	751
22:00	36	54	37	23	32	39	0	14	8	51	8	0	15	13	7	8	17	0	0	12	23	8	0	12	28	3	0	14	15	0	15	0	16	507
23:00	11	10	0	0	0	0	0	0	0	8	0	0	0	0	0	0	0	0	0	0	0	0	0	0	15	10	0	0	0	0	0	0	0	54

Supplementary Discussion 4: Service reduction during disruption

In Supplementary Table 15, the service reduction of the Robust Model with $k = 1$ under two charging stations disruption ($r = 2$) is detailed. The total number of possible failure combinations is 153 ($^{18}C_2$), with most of them (121 combinations, ≈79%) producing zero service reduction. The remaining failure scenarios (32 combinations) could reduce the service by up to a maximum of 13.73%.

Similarly, Supplementary Table 16 shows the service reduction of the Robust Model with $k = 1$ under three charging stations disruption ($r = 3$). Overall, there are 816 possible failure combinations ($^{18}C_3$), and only 376 (≈46%) combinations will result in service reductions with a maximum of 22.38%.

In Supplementary Table 17, the service reduction of the Robust Model with $k = 2$ under three charging stations disruption ($r = 3$) is illustrated. From 5,456 ($^{33}C_3$) possible failure combinations, only 50 (≈0.9%) combinations have non-zero service reductions with a maximum of 8.92%.

Lastly, Supplementary Table 18 compares the performance of the three models during disruption ($r = 1, 2,$ and 3).

Supplementary Table 15. Service reduction at $r = 2$ (Robust Model with $k = 1$)

Disrupted stations	1	3	4	7	9	10	11	14	16	19	21	24	30	31	33	35	47	72
1	NA	10.8	10.18	3.55	4.57	0	4.03	0	0	0	0.72	1.88	7.78	4.69	10.89	2.49	2.44	0
3			0	3.08	0	0	0	0	1.88	0	0	0	0	0	13.73	0	2.94	0
4				6.58	1.54	2.19	6.99	0	0	8.76	5.72	0	0	2.76	0	0	0	1.43
7					0	0	0	0	0	0	0	0	0	0	3.03	0	0	0
9						0	5.84	0	0	0	0	0	0	0	0	0	0	0
10							0	0	0	0	0	0	0	0	0	0	0	0
11								0	0	0	0	0	4	6.25	0	3.94	0	0.14
14									0	0	0.84	0	0	0	0	0	0	0
16										0	0	0	0	0	0	0	0	0
19											0	0	0	0	0	0	0	0
21												0	0	0	0	0	0	0
24													0	0	0	0	0	0
30														4.98	0	0	0	0
31															0	0	0	0
33																0	0	0
35																	0	0
47																		0
72																		NA

Supplementary Table 16. Service reduction at $r = 3$ (Robust Model with $k = 1$)

Disrupted stations	Service reduction	Disrupted stations	Service reduction	Disrupted stations	Service reduction	Disrupted stations	Service reduction	Disrupted stations	Service reduction	Disrupted stations	Service reduction
(1, 3, 4)	22.38%	(1, 10, 31)	4.68%	(1, 35, 72)	2.63%	(3, 30, 47)	2.96%	(4, 16, 21)	5.72%	(9, 11, 72)	6.06%
(1, 3, 7)	10.79%	(1, 10, 33)	10.39%	(1, 47, 72)	2.44%	(3, 31, 33)	13.67%	(4, 16, 31)	2.76%	(9, 14, 21)	0.84%
(1, 3, 9)	14.67%	(1, 10, 35)	2.60%	(3, 4, 7)	9.93%	(3, 31, 47)	2.94%	(4, 16, 72)	1.43%	(9, 30, 31)	4.95%
(1, 3, 10)	10.97%	(1, 10, 47)	2.44%	(3, 4, 9)	1.43%	(3, 33, 35)	13.83%	(4, 19, 21)	8.87%	(10, 11, 30)	4.07%
(1, 3, 11)	14.96%	(1, 11, 14)	3.98%	(3, 4, 10)	2.24%	(3, 33, 47)	13.64%	(4, 19, 24)	8.96%	(10, 11, 31)	6.47%
(1, 3, 14)	10.54%	(1, 11, 16)	3.96%	(3, 4, 11)	6.99%	(3, 33, 72)	13.62%	(4, 19, 30)	9.28%	(10, 11, 35)	3.67%
(1, 3, 16)	13.28%	(1, 11, 19)	3.89%	(3, 4, 16)	1.88%	(3, 35, 47)	2.96%	(4, 19, 31)	11.47%	(10, 11, 72)	0.14%
(1, 3, 19)	11.09%	(1, 11, 21)	3.94%	(3, 4, 19)	9.07%	(3, 47, 72)	2.97%	(4, 19, 33)	9.14%	(10, 14, 21)	0.84%
(1, 3, 21)	11.23%	(1, 11, 24)	6.02%	(3, 4, 21)	5.70%	(4, 7, 9)	8.69%	(4, 19, 35)	8.74%	(10, 30, 31)	5.27%
(1, 3, 24)	12.87%	(1, 11, 30)	13.53%	(3, 4, 31)	2.72%	(4, 7, 10)	8.82%	(4, 19, 47)	9.17%	(11, 14, 21)	0.84%
(1, 3, 30)	18.98%	(1, 11, 31)	11.75%	(3, 4, 33)	13.62%	(4, 7, 11)	13.56%	(4, 19, 72)	15.25%	(11, 14, 30)	4.03%
(1, 3, 31)	15.66%	(1, 11, 33)	15.27%	(3, 4, 47)	2.92%	(4, 7, 14)	6.58%	(4, 21, 24)	5.00%	(11, 14, 31)	6.24%
(1, 3, 33)	16.75%	(1, 11, 35)	5.93%	(3, 4, 72)	2.80%	(4, 7, 16)	6.58%	(4, 21, 30)	5.72%	(11, 14, 35)	3.71%
(1, 3, 35)	13.19%	(1, 11, 47)	6.47%	(3, 7, 9)	3.06%	(4, 7, 19)	9.57%	(4, 21, 31)	8.48%	(11, 14, 72)	0.16%
(1, 3, 47)	13.37%	(1, 11, 72)	4.03%	(3, 7, 10)	3.06%	(4, 7, 21)	6.25%	(4, 21, 33)	5.72%	(11, 16, 30)	4.01%
(1, 3, 72)	11.36%	(1, 14, 21)	1.56%	(3, 7, 11)	3.17%	(4, 7, 24)	9.19%	(4, 21, 35)	5.72%	(11, 16, 31)	6.24%
(1, 4, 7)	16.91%	(1, 14, 24)	2.26%	(3, 7, 14)	3.12%	(4, 7, 30)	6.58%	(4, 21, 47)	5.72%	(11, 16, 35)	3.69%
(1, 4, 9)	17.56%	(1, 14, 30)	7.79%	(3, 7, 16)	5.00%	(4, 7, 31)	9.34%	(4, 21, 72)	7.15%	(11, 16, 72)	0.16%
(1, 4, 10)	12.76%	(1, 14, 31)	4.37%	(3, 7, 19)	2.97%	(4, 7, 33)	9.89%	(4, 24, 31)	2.76%	(11, 19, 30)	3.98%
(1, 4, 11)	19.28%	(1, 14, 33)	10.39%	(3, 7, 21)	3.01%	(4, 7, 35)	6.58%	(4, 24, 72)	1.43%	(11, 19, 31)	6.33%
(1, 4, 14)	10.68%	(1, 14, 35)	2.37%	(3, 7, 24)	3.01%	(4, 7, 47)	6.58%	(4, 30, 31)	7.63%	(11, 19, 35)	3.87%
(1, 4, 16)	10.18%	(1, 14, 47)	2.44%	(3, 7, 30)	3.08%	(4, 7, 72)	7.88%	(4, 30, 72)	1.43%	(11, 19, 72)	0.14%
(1, 4, 19)	21.81%	(1, 16, 21)	0.72%	(3, 7, 31)	3.17%	(4, 9, 10)	3.46%	(4, 31, 33)	2.76%	(11, 21, 30)	4.09%
(1, 4, 21)	16.61%	(1, 16, 24)	1.88%	(3, 7, 33)	14.24%	(4, 9, 11)	9.62%	(4, 31, 35)	2.76%	(11, 21, 31)	6.11%
(1, 4, 24)	12.44%	(1, 16, 30)	8.13%	(3, 7, 35)	3.24%	(4, 9, 14)	1.63%	(4, 31, 47)	2.76%	(11, 21, 35)	3.89%
(1, 4, 30)	21.02%	(1, 16, 31)	4.44%	(3, 7, 47)	6.13%	(4, 9, 16)	1.51%	(4, 31, 72)	5.88%	(11, 21, 72)	0.14%
(1, 4, 31)	18.08%	(1, 16, 33)	10.32%	(3, 7, 72)	3.12%	(4, 9, 19)	9.64%	(4, 33, 72)	2.29%	(11, 24, 30)	3.98%
(1, 4, 33)	18.40%	(1, 16, 35)	2.58%	(3, 9, 11)	6.45%	(4, 9, 21)	7.81%	(4, 35, 72)	1.43%	(11, 24, 31)	6.00%
(1, 4, 35)	13.26%	(1, 16, 47)	2.44%	(3, 9, 16)	1.86%	(4, 9, 24)	1.90%	(4, 47, 72)	1.43%	(11, 24, 35)	4.10%
(1, 4, 47)	12.61%	(1, 19, 21)	0.72%	(3, 9, 33)	13.65%	(4, 9, 30)	1.51%	(7, 9, 11)	5.73%	(11, 24, 72)	0.14%
(1, 4, 72)	16.47%	(1, 19, 24)	1.88%	(3, 9, 47)	2.92%	(4, 9, 31)	4.53%	(7, 9, 33)	3.05%	(11, 30, 31)	7.10%
(1, 7, 9)	7.36%	(1, 19, 30)	7.90%	(3, 10, 16)	1.86%	(4, 9, 33)	1.43%	(7, 10, 33)	2.96%	(11, 30, 33)	3.98%
(1, 7, 10)	3.55%	(1, 19, 31)	4.80%	(3, 10, 33)	13.58%	(4, 9, 35)	1.43%	(7, 11, 30)	4.03%	(11, 30, 35)	7.79%
(1, 7, 11)	7.36%	(1, 19, 33)	10.59%	(3, 10, 47)	2.94%	(4, 9, 47)	1.68%	(7, 11, 31)	6.07%	(11, 30, 47)	4.23%
(1, 7, 14)	3.60%	(1, 19, 35)	2.40%	(3, 11, 16)	1.88%	(4, 9, 72)	3.55%	(7, 11, 33)	3.03%	(11, 30, 72)	3.98%
(1, 7, 16)	3.74%	(1, 19, 47)	2.44%	(3, 11, 30)	3.96%	(4, 10, 11)	9.01%	(7, 11, 35)	3.83%	(11, 31, 33)	6.29%
(1, 7, 19)	3.57%	(1, 19, 72)	1.33%	(3, 11, 31)	6.06%	(4, 10, 14)	2.13%	(7, 11, 72)	0.16%	(11, 31, 35)	10.25%
(1, 7, 21)	3.85%	(1, 21, 24)	2.53%	(3, 11, 33)	13.81%	(4, 10, 16)	2.06%	(7, 14, 21)	0.84%	(11, 31, 47)	6.09%
(1, 7, 24)	5.46%	(1, 21, 30)	8.55%	(3, 11, 35)	3.66%	(4, 10, 19)	10.61%	(7, 14, 33)	3.06%	(11, 31, 72)	6.07%
(1, 7, 30)	12.20%	(1, 21, 31)	4.80%	(3, 11, 47)	2.96%	(4, 10, 21)	7.94%	(7, 16, 33)	2.99%	(11, 33, 35)	3.66%
(1, 7, 31)	8.13%	(1, 21, 33)	11.22%	(3, 11, 72)	0.14%	(4, 10, 24)	2.11%	(7, 19, 24)	2.24%	(11, 33, 72)	0.16%
(1, 7, 33)	10.37%	(1, 21, 35)	2.78%	(3, 14, 16)	1.88%	(4, 10, 30)	2.24%	(7, 19, 33)	2.99%	(11, 35, 47)	3.98%
(1, 7, 35)	6.25%	(1, 21, 47)	2.60%	(3, 14, 21)	0.86%	(4, 10, 31)	2.76%	(7, 21, 33)	3.01%	(11, 35, 72)	3.71%
(1, 7, 47)	6.02%	(1, 21, 72)	0.72%	(3, 14, 33)	13.67%	(4, 10, 33)	2.24%	(7, 24, 33)	3.14%	(11, 47, 72)	0.14%
(1, 7, 72)	3.55%	(1, 24, 30)	9.82%	(3, 14, 47)	2.92%	(4, 10, 35)	2.06%	(7, 30, 31)	5.07%	(14, 16, 21)	0.84%
(1, 9, 10)	4.68%	(1, 24, 31)	6.49%	(3, 16, 19)	1.86%	(4, 10, 47)	2.08%	(7, 30, 33)	3.05%	(14, 19, 21)	0.86%
(1, 9, 11)	14.16%	(1, 24, 33)	12.63%	(3, 16, 21)	1.86%	(4, 10, 72)	3.67%	(7, 31, 33)	3.05%	(14, 21, 24)	0.86%
(1, 9, 14)	3.49%	(1, 24, 35)	4.21%	(3, 16, 24)	1.88%	(4, 11, 14)	6.99%	(7, 33, 35)	2.99%	(14, 21, 30)	0.84%
(1, 9, 16)	3.46%	(1, 24, 47)	4.28%	(3, 16, 30)	1.86%	(4, 11, 16)	6.99%	(7, 33, 47)	3.01%	(14, 21, 31)	0.86%
(1, 9, 19)	5.03%	(1, 24, 72)	1.88%	(3, 16, 31)	1.88%	(4, 11, 19)	14.82%	(7, 33, 72)	3.03%	(14, 21, 33)	0.86%
(1, 9, 21)	3.66%	(1, 30, 31)	9.34%	(3, 16, 33)	15.32%	(4, 11, 21)	12.70%	(9, 10, 11)	5.93%	(14, 21, 35)	0.07%
(1, 9, 24)	6.13%	(1, 30, 33)	18.63%	(3, 16, 35)	1.88%	(4, 11, 24)	6.99%	(9, 10, 31)	2.01%	(14, 21, 47)	0.84%
(1, 9, 30)	8.60%	(1, 30, 35)	12.72%	(3, 16, 47)	5.05%	(4, 11, 30)	10.89%	(9, 11, 14)	5.68%	(14, 21, 72)	0.88%
(1, 9, 31)	9.08%	(1, 30, 47)	10.97%	(3, 16, 72)	1.86%	(4, 11, 31)	11.32%	(9, 11, 16)	5.77%	(14, 30, 31)	5.23%
(1, 9, 33)	13.94%	(1, 30, 72)	8.06%	(3, 19, 33)	13.67%	(4, 11, 33)	6.99%	(9, 11, 19)	5.64%	(16, 30, 31)	4.98%
(1, 9, 35)	6.86%	(1, 31, 33)	14.96%	(3, 19, 47)	2.96%	(4, 11, 35)	10.55%	(9, 11, 21)	5.86%	(19, 30, 31)	4.96%
(1, 9, 47)	5.91%	(1, 31, 35)	6.81%	(3, 21, 33)	13.80%	(4, 11, 47)	6.99%	(9, 11, 24)	5.64%	(21, 30, 31)	5.39%
(1, 9, 72)	3.73%	(1, 31, 47)	6.84%	(3, 21, 47)	2.96%	(4, 11, 72)	8.58%	(9, 11, 30)	9.60%	(24, 30, 31)	5.03%
(1, 10, 11)	6.58%	(1, 31, 72)	4.41%	(3, 24, 33)	13.65%	(4, 14, 19)	8.85%	(9, 11, 31)	11.83%	(30, 31, 33)	4.93%
(1, 10, 14)	0.52%	(1, 33, 35)	12.79%	(3, 24, 47)	2.96%	(4, 14, 21)	6.59%	(9, 11, 33)	5.70%	(30, 31, 35)	5.02%
(1, 10, 21)	0.72%	(1, 33, 47)	16.20%	(3, 30, 31)	5.30%	(4, 14, 31)	2.76%	(9, 11, 35)	9.59%	(30, 31, 47)	5.14%
(1, 10, 24)	1.88%	(1, 33, 72)	10.41%	(3, 30, 33)	13.64%	(4, 14, 72)	1.43%	(9, 11, 47)	5.64%	(30, 31, 72)	4.91%
(1, 10, 30)	7.81%	(1, 35, 47)	5.09%	(3, 30, 35)	1.94%	(4, 16, 19)	8.51%				

Supplementary Table 17. Service reduction at $r = 3$ (Robust Model with $k = 2$)

Disrupted stations	Service reduction	Disrupted stations	Service reduction	Disrupted stations	Service reduction	Disrupted stations	Service reduction	Disrupted stations	Service reduction	Disrupted stations	Service reduction
(1, 2, 3)	3.58%	(1, 11, 27)	3.08%	(1, 30, 38)	3.53%	(3, 33, 39)	0.84%	(4, 11, 31)	2.10%	(4, 31, 50)	2.01%
(1, 2, 7)	3.60%	(1, 11, 30)	5.39%	(1, 33, 47)	0.27%	(4, 7, 18)	1.77%	(4, 17, 19)	0.43%	(6, 14, 22)	1.47%
(1, 2, 33)	8.92%	(1, 11, 31)	1.00%	(1, 47, 48)	1.06%	(4, 7, 19)	4.89%	(4, 18, 19)	0.47%	(7, 19, 21)	1.79%
(1, 3, 33)	4.16%	(1, 11, 35)	3.12%	(2, 3, 7)	3.05%	(4, 7, 21)	4.96%	(4, 18, 21)	4.55%	(11, 27, 35)	2.99%
(1, 3, 39)	3.14%	(1, 22, 24)	1.09%	(2, 3, 33)	8.40%	(4, 9, 10)	1.72%	(4, 19, 21)	5.21%	(11, 30, 31)	4.39%
(1, 3, 47)	0.25%	(1, 27, 35)	1.00%	(2, 7, 33)	3.05%	(4, 9, 43)	1.77%	(4, 19, 24)	3.28%	(11, 30, 35)	1.52%
(1, 4, 6)	5.52%	(1, 28, 30)	4.23%	(3, 15, 16)	1.88%	(4, 10, 31)	2.06%	(4, 19, 25)	4.28%	(15, 16, 17)	1.83%
(1, 4, 25)	6.13%	(1, 30, 31)	4.82%	(3, 16, 17)	1.86%	(4, 10, 43)	1.13%	(4, 28, 31)	2.58%	(28, 30, 31)	4.39%
(1, 6, 30)	2.58%	(1, 30, 35)	1.94%								

Supplementary Table 18. Maximum service reductions for all models

Model	Base Model	Robust Model, $k = 1$	Robust Model, $k = 2$
Maximum service reduction if one station fails ($r = 1$)	34.03%	0.00%	0.00%
Maximum service reduction if two stations fail ($r = 2$)	58.18%	13.73%	0.00%
Maximum service reduction if three stations fail ($r = 3$)	59.67%	22.38%	8.92%

Supplementary Discussion 5: Case Study Two - Guelph Transit

The proposed BEB system configuration model is applied to a multiple-hub transit network in Guelph City, Ontario, Canada. The network comprises 23 routes served by 55 buses and 506 bus stops/terminals. The Guelph network has 18 transfer stations and terminals, serving multiple routes and long recovery times. These locations are selected as the candidate charging stations, illustrated in Supplementary Fig. 4. More details about the network timetable are presented in the work of Foda [31].

The solution algorithm converges in four iterations after adding nine failure scenarios. The results of the system configurations of the Base Model and the Robust Model with $k = 1$ are presented in Supplementary Table 19. The optimal BEB system under nominal operation requires ten heterogeneous charging stations equipped with 14 poles. The main station is Station ID 4, which comprises a 1000 kW charger unit with four poles. The disruption of this station leads to 20 failed buses (36% of the total number of buses). In comparison, the resilient model that could fulfill all the operational trips under one station disruption (Robust Model, $k = 1$) requires 17 charging stations equipped with 23 poles.

In Supplementary Table 20, the distribution of the annual system costs of the two models is illustrated. In this network, the price of the robustness (PoR) of the robust BEB system configurations with $k = 1$ is 4.69%. This will prevent the failure of up to 20 BEBs in case of one station failure.

Comparing the results of Oakville and Guelph transit networks emphasizes that the impact of the charging station disruption in the transit network and the price of robustness are functions of multiple factors, such as the spatial configuration of the network (network type and graph density), number and specification of the buses, location of the charging stations, number and specifications of the charging stations, the charging schedule, and the operational timetable.

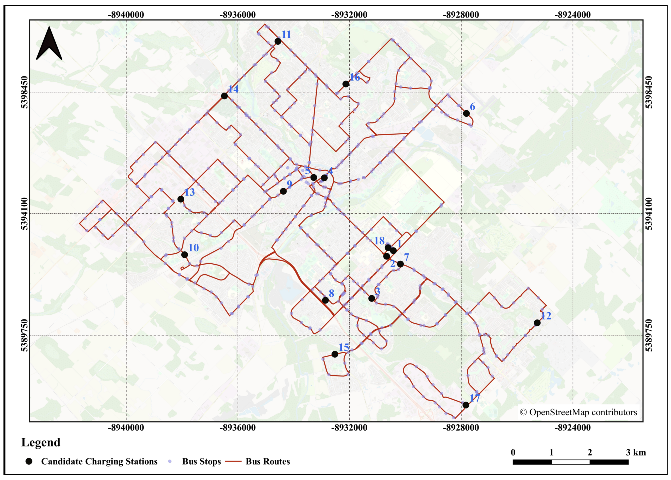

Supplementary Fig. 4 Candidate charging stations of the Guelph network

Legend: The map illustrates the transit network routes as red lines, bus stops as small blue dots, and locations of the candidate charging stations are marked in black dots numbered from 1 to 18. The maps are drawn using QGIS [26] used under the GNU General Public License Version 2 [27]. The GTFS data for Guelph City used to draw the transit network elements is publicly available [32] and used under the City of Guelph Open Government Licence [33]. The OpenStreetMap is used in the maps under the Open Data Commons Open Database License (ODBL) by the OpenStreetMap Foundations (OSMF) [30].

Supplementary Table 19. Results of BEB system configuration (Base and Robust Model)

Models	Total annual system cost ($/year)	Number of buses (#) × Battery size (kWh)	Number of charging stations	Power of charger units (kW)	Number of poles (#)
Base Model	$ 4,763,136.459	51×100<br>3×200<br>1×600	10	8×250<br>1×500<br>1×1000	14
Robust Model<br>$k = 1$	$ 4,986,729.762	35×100<br>17×200<br>2×400<br>1×600	17	6×250<br>11×500	23

Supplementary Table 20. System annual costs (Base and Robust Model)

Parameter	Base Model	Robust Model, $k = 1$
Infrastructure cost	$ 79,914.130	$ 135,854.020
Chargers cost	$ 131,272.277	$ 255,618.662
Battery cost	$ 335,639.344	$ 442,191.517
Fleet cost	$ 3,223,203.224	$ 3,223,203.224
Capital costs	$ 3,770,028.974	$ 4,056,867.423
Operational costs*	$ 993,107.485	$ 929,862.339
Total annual cost	$ 4,763,136.459	$ 4,986,729.762

* Operational costs include the electricity ToU and demand charges, and emissions costs and are estimated in the Robust Model based on the scenario of no charging station failures. Please note that the battery capacity of each model is a decision variable, hence the variation of the battery cost across models.

Supplementary Discussion 6: Sensitivity Analysis

Sensitivity analyses of some key parameters, including the cost of the charging system (infrastructure and chargers), battery cost, and operational cost (electricity ToU, demand charges, and GHG emissions), are conducted for the robust model with $k = 1$. In each case, four additional scenarios are solved. These scenarios are generated by multiplying the base cost parameter with 0.5, 0.75, 1.25, and 1.5.

Supplementary Fig. 5 illustrates the change in the total annual system costs associated with the variation in the charging system cost (from 0.5 to 1.5 times the base cost). Moreover, the BEB system configuration in each scenario is presented in Supplementary Table 21. The results indicate that increasing the charging cost will increase the system cost, which is logical. However, both are not growing at the same rate. In other words, with a multiplied factor equal to 0.5, the charging system cost and the total system cost of a Robust model with k = 1 are $255,671 and $6,996,530, respectively. After increasing the factor to 1.5 (300%), the charging system cost and the total system cost are increased by 259,131 and $7,313,724, respectively. The reason is the trade-off between the charging systems, battery, and operational costs.

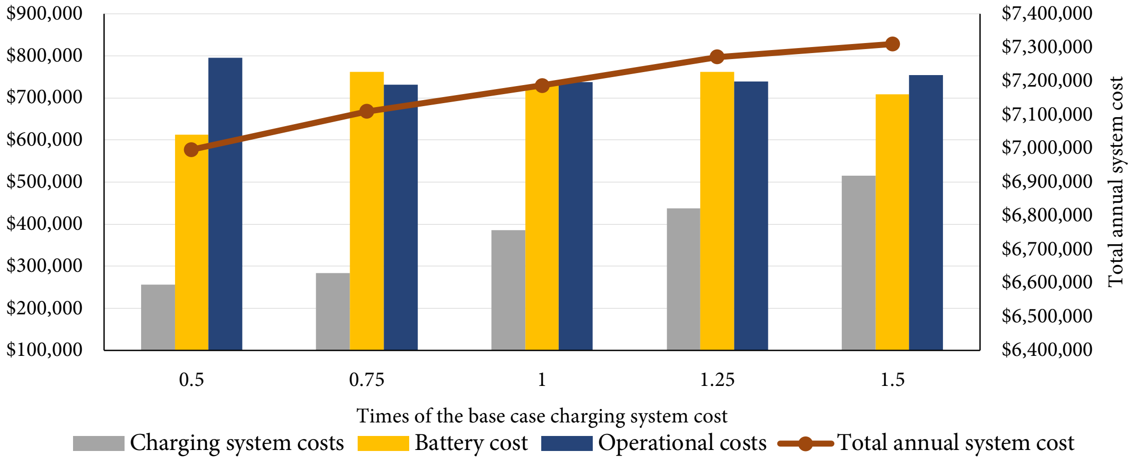

Supplementary Fig. 5 The BEB system costs under various charging system costs.

Legend: The figure describes the sensitivity of the three main cost components in the BEB system (the charging system, battery, and operating) and the total annual system cost to the charger cost. The x-axis presents the factor value multiplied by the base charger cost to estimate the scenarios. In total, five scenarios are presented with factor values ranging from 0.5 to 1.5 with 0.25 increments. In each scenario, the values of the three main cost components are shown as bars, and the total annual system cost is illustrated as a brown line.

Supplementary Table 21. Results of BEB system configuration under various charging system costs (Robust Models, $k = 1$)

Factor	Price of Robustness (PoR %)	Additional cost to the Base Model ($)	Number of buses (#) × Battery size (kWh)			Number of charging stations	Power of charger units (kW)	Number of poles (#)
0.5	1.34%	$92,769.151	69×100	20×200	2×300	22	6×250<br>16×500	23
0.75	1.91%	$133,198.279	48×100<br>39×200	2×300<br>1×500	1×600	17	7×250<br>10×500	19
1	3.26%	$227,207.206	54×100<br>33×200	2×300<br>1×500	1×600	18	9×250<br>9×500	19
1.25	3.26%	$229,431.065	54×100<br>29×200	5×300<br>2×500	1×600	15	4×250<br>11×500	16
1.5	3.56%	$250,965.070	52×100	36×200	3×300	16	8×250<br>8×500	17

The same observation is associated with the variation in battery cost (Supplementary Fig. 6 and Supplementary Table 22). Increasing the battery price leads to a decrease in the number of large battery sizes to achieve a minimum total annual system cost. Therefore, the optimization model seeks to deploy more charging stations and increase operational costs by considering a more frequent charging schedule. An optimal trade-off between all the system components achieves the minimum total annual system cost. The PoR is increasing with the increase of the multiplier factor to the battery price parameter. This is attributed to the fact that one of the main approaches to achieving a robust model against charging station failure is to increase BEB battery size (first-stage variables). Therefore, with an increased battery price, the Base

Model will choose lower battery capacities. However, the Robust Model will increase the selected battery capacities, leading to a larger difference between the Base Model and the Robust Model (increase the PoR).

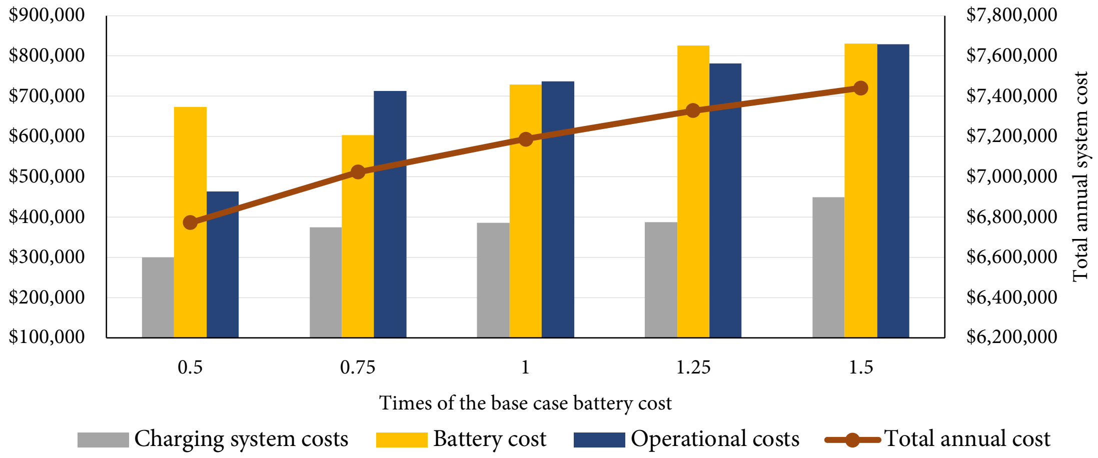

Supplementary Fig. 6 The BEB system costs under various battery costs.

Legend: The figure describes the sensitivity of the three main cost components in the BEB system (the charging system, battery, and operating) and the total annual system cost to the battery cost. The x-axis presents the factor value multiplied by the base battery cost to estimate the scenarios. In total, five scenarios are presented with factor values ranging from 0.5 to 1.5 with 0.25 increments. In each scenario, the values of the three main cost components are shown as bars, and the total annual system cost is illustrated as a brown line.

Supplementary Table 22. Results of BEB system configuration under various battery costs (Robust Models, $k = 1$)

Factor	Price of Robustness (PoR %)	Additional cost to the Base Model ($)	Number of buses (#) × Battery size (kWh)			Number of charging stations	Power of charger units (kW)	Number of poles (#)
0.5	2.80%	$184,617.775	20×100<br>24×200	22×300<br>12×400	8×500<br>4×600<br>1×700	14	7×250<br>7×500	15
0.75	2.83%	$193,714.218	46×100<br>37×200	5×300<br>2×500	1×600	17	7×250<br>10×500	17
1	3.26%	$227,207.206	54×100<br>33×200	2×300<br>1×500	1×600	18	9×250<br>9×500	19
1.25	3.32%	$237,154.164	66×100<br>22×200	1×300<br>1×500	1×600	19	12×250<br>7×500	20
1.5	3.36%	$241,690.481	81×100<br>8×200	1×300<br>1×400		23	13×250<br>10×500	24

The impacts of varying the cost of operational parameters (electricity ToU, demand charges, and GHG emissions) from 0.5 to 1.5 times the base prices on total system costs and configuration are presented in Supplementary Fig. 7 and Supplementary Table 23. Increasing operational costs would result in a higher total system cost. However, a trade-off emerges among system components (charging system, battery capacities, and charging schedule) to optimize total system costs under increased operational costs.

These results indicate that when the operational cost parameters increase, the strategy involves enlarging battery sizes for BEBs to reduce charging demand during on- and mid-peak periods. What truly stands out is the reduced number of charging stations as operational cost parameters continue to rise. This reduction can be attributed to including demand charge costs, estimated individually for each allocated station. Another notable trend is the decreasing PoR as the operational cost parameters grow, in contrast to previous cases. Since system operational costs are linked to the charging schedule (second-stage variables), increasing these parameters leads to larger first-stage variables (charging system and battery sizes). Consequently, the base model becomes more robust, resulting in a smaller difference between the Robust Model and the Base Model (PoR) as the operational cost parameters increase.

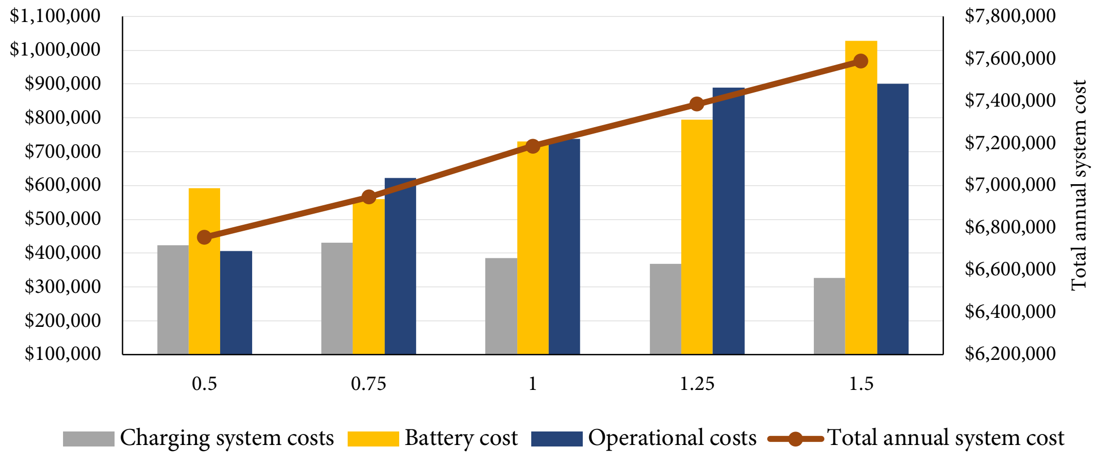

Supplementary Fig. 7 The BEB system costs under various operational parameters costs.

Legend: The figure describes the sensitivity of the three main cost components in the BEB system (the charging system, battery, and operating) and the total annual system cost to the operational costs. The x-axis presents the factor value multiplied by the base operational costs to estimate the scenarios. In total, five scenarios are presented with factor values ranging from 0.5 to 1.5 with 0.25 increments. In each scenario, the values of the three main cost components are shown as bars, and the total annual system cost is illustrated as a brown line.

Supplementary Table 23. Results of BEB system configuration under various operational parameters costs (Robust Models, $k = 1$)

Factor	Price of Robustness (PoR %)	Additional cost to the Base Model ($)	Number of buses (#) × Battery size (kWh)			Number of charging stations	Power of charger units (kW)	Number of poles (#)
0.5	3.81%	$248,017.696	72×100	18×200	1×300	22	17×250<br>5×500	23
0.75	3.47%	$235,610.607	77×100	14×200		22	16×250<br>6×500	23
1	3.26%	$227,207.206	54×100<br>33×200	2×300<br>1×500	1×600	18	9×250<br>9×500	19
1.25	2.79%	$200,311.778	43×100<br>43×200	3×300<br>1×500	1×600	17	8×250<br>9×500	18
1.5	2.04%	$151,632.156	20×100<br>53×200	11×300<br>3×400	2×500<br>2×600	16	10×250<br>6×500	16

In closure, the sensitivity analyses' results indicate that no dominant variables impact the outputs of the model. This is mainly attributed to the intertwined dynamic relationships between the BEB system components.

Supplementary Discussion 7: Solution Algorithms

Algorithm 1 Outer-Level C&CG Algorithm Framework

Initialize: $\varepsilon \geq 0, n = 0, UB = \infty, LB = -\infty$, and ξ_i^0 s. t. $\xi \in \Xi$.
while $G_O = \left|\frac{UB-LB}{UB}\right| > \varepsilon$ **do**
Solve the MP in (23-40).
Save the first-stage variables solution $(x^n, P^{st,n}, N^{po,n}, Q^n)$ and objective function value F_1^n.
Update LB= F_1^n.
Take $\hat{x} = x^n, \hat{P}^{st} = P^{st,n}, \hat{N}^{po} = N^{po,n}, \hat{Q} = Q^n$, and solve the SP in (41-50).
Get the charging station failure worst-case scenario ξ^{n+1}.
Get the worst-case cost F_2^n of the SP.
Update the UB = $\min\{UB, F_2^n + \tau \sum_{i\in I} \rho^{st}\hat{x}_i + \tau \sum_{i\in I}(\hat{P}_i^{st}\rho^{ch} + \hat{N}_i^{po}\rho^{po}) + \tau \sum_{b\in B}(\rho^{batt}\hat{Q}_b + \rho^{bus})\}$.
Add new variables $z^{n+1}, y^{n+1}, \alpha^{n+1}, \gamma^{n+1}, P^{n+1}, P^{d,n+1}, P^{avg,n+1}, S^{dep,n+1}$ and Constraints (24-39) to the MP.
Set $n := n + 1$.
end while
return the optimal robust BEB system configuration $x^n, P^{st,n}, N^{po,n}, Q^n$, and the UB value.

Algorithm 2 Inner-Level C&CG Algorithm Framework

Initialize: $\varepsilon \geq 0, s = 0, IUB = \infty, ILB = -\infty$.
while $G_I = \left|\frac{IUB-ILB}{IUB}\right| > \varepsilon$ **do**
Solve the IMP in (70-74).
Save the optimal solution of the random variable (ξ^s) and objective function value f_1^s.
Update IUB= f_1^s.
Take $\hat{\xi} = \xi^s$, and solve the ISP in (75-77).
Get the optimal solution of the second-stage discrete variables $(z^{s+1}, y^{s+1}, \alpha^{s+1}, \gamma^{s+1})$.
Get the worst-case cost f_2^s of the ISP.
Update the ILB = $\max\{ILB, f_2^s\}$.
Add new continuous variables $P^{s+1}, P^{d,s+1}, P^{avg,s+1}, S^{dep,s+1}$ and Constraints (71-74) to the IMP.
Set $s := s + 1$.
end while
return the worst-case scenario $\xi^n := \hat{\xi}$ to the MP

Supplementary Discussion 8: Computational performance enhancement

During the process of the standard C&CG algorithm (outer and inner levels), the number of variables and constraints will increase quickly over iterations in the master problem. Therefore, this subsection details three computational performance enhancement strategies implemented to reduce the solution framework time.

The first approach is to add multiple scenarios in each iteration of the outer-level problem. In the column-and-constrained generation (C&CG) framework, the sub-problem supplies the master problem with the worst-case scenario under the provided values of the first-stage variables. Instead of adding a single optimal scenario, multiple significant scenarios could be added to the master problem to speed up the increase of the lower bound and reduce the number of times the master problem is solved. The first added scenario is the worst-case one. The other scenarios could be estimated by solving the sub-problem after adding feasibility cuts that remove the existing scenarios in the master problem or the addition set in this iteration. To avoid adding unnecessary scenarios to the master problem, we recommend using this approach in the first iterations and decreasing the number of added scenarios through iterations until it reaches the basic process (one scenario).

The dimensions of the inner master problem increase dramatically through iterations of the inner-level C&CG solution framework. Accordingly, the increase in the inner-level problem solution time could be due to the time of solving the final iterations of the inner master problem. The second approach to enhance the computational performance is to replace the inner-master problem with an enumeration algorithm that generates all the feasible scenarios of the charging station's failures (ξ). Then, the inner sub-problem will be solved in all these scenarios, and the worst-case one or multiple scenarios will be chosen (highest objective function). Several constraints could be used to reduce the number of enumerated scenarios, such as the random failure variable of a location is one only if a charging station is built in this location in the master problem. In addition, the added scenarios in the master problem until this iteration are not considered. We recommend using this approach only in cases of small failure uncertainty budget (k).

Through the solution iterations, more charging station failure scenarios will be added to the master problem, increasing the BEB system cost (LB) due to increasing the BEB system robustness (reducing the unsatisfied operational trips in charging station failure cases). From another viewpoint, this process could be handled as a multi-objective optimization problem that investigates the trade-off between the BEB system configuration cost and the system robustness. As such, another termination criteria could be added to the outer-level C&CG framework. This criterion ends the algorithm earlier if the decision maker is satisfied with the expected worst-case unserved ratio of the operational trips and cannot invest additional money in increasing the system's robustness. This trade-off is different than the trade-off between the budget number of charging station failures (k) and the system costs.

Supplementary References

1. Derek. Life by Numbers Explaining the Electricity Sector in Ontario. 2023. https://www.lifebynumbers.ca/history/ottawa-blackouts/. (2023)

2. Outage list of BC Hydro Power smart. 2023. https://www.bchydro.com/power-outages/app/outage-list-planned.html#planned. (2023)

3. Wilson K. Strong winds cause power outages across the GTA. 2022. https://www.cp24.com/news/strong-winds-cause-power-outages-across-the-gta-1.6179614. (2023)

4. Mukherjee S, Nateghi R, Hastak M. Data on major power outage events in the continental U.S. *Data Brief* **19**, 2079-2083 (2018).

5. Do V, *et al.* Spatiotemporal distribution of power outages with climate events and social vulnerability in the USA. *Nat Commun* **14**, 2470 (2023).

6. Raman G, Raman G, Peng JC. Resilience of urban public electric vehicle charging infrastructure to flooding. *Nat Commun* **13**, 3213 (2022).

7. Zhou Y, Ong GP, Meng Q, Cui HP. Electric bus charging facility planning with uncertainties: Model formulation and algorithm design. *Transportation Research Part C-Emerging Technologies* **150**, (2023).

8. Gairola P, Nezamuddin N. Optimization framework for integrated battery electric bus planning and charging scheduling. *Transportation Research Part D-Transport and Environment* **118**, (2023).

9. Liu K, Gao H, Wang Y, Feng T, Li C. Robust charging strategies for electric bus fleets under energy consumption uncertainty. *Transportation Research Part D-Transport and Environment* **104**, (2022).

10. Zhou Y, Wang H, Wang Y, Li R. Robust optimization for integrated planning of electric-bus charger deployment and charging scheduling. *Transportation Research Part D-Transport and Environment* **110**, (2022).

11. Hu H, Du B, Liu W, Perez P. A joint optimisation model for charger locating and electric bus charging scheduling considering opportunity fast charging and uncertainties. *Transportation Research Part C-Emerging Technologies* **141**, (2022).

12. Bai ZJ, Yang L, Fu CY, Liu ZC, He ZB, Zhu N. A robust approach to integrated wireless charging infrastructure design and bus fleet size optimization. *Computers & Industrial Engineering* **168**, (2022).

13. Kong L, Zhang H, Li W, Bai H, Dai N. Spatial–Temporal Scheduling of Electric Bus Fleet in Power-Transportation Coupled Network. *IEEE Transactions on Transportation Electrification* **9**, 2969-2982 (2023).

14. Zheng FF, Wang ZJ, Liu M. Overnight charging scheduling of battery electric buses with uncertain charging time. *Operational Research* **22**, 4865-4903 (2022).

15. Iliopoulou C, Kepaptsoglou K. Robust electric transit route network design problem (RE-TRNDP) with delay considerations: Model and application. *Transportation Research Part C-Emerging Technologies* **129**, (2021).

16. An K. Battery electric bus infrastructure planning under demand uncertainty. *Transportation Research Part C-Emerging Technologies* **111**, 572-587 (2020).

17. Liu ZC, Song ZQ, He Y. Planning of Fast-Charging Stations for a Battery Electric Bus System under Energy Consumption Uncertainty. *Transportation Research Record* **2672**, 96-107 (2018).

18. Liu ZC, Song ZQ. Robust planning of dynamic wireless charging infrastructure for battery electric buses. *Transportation Research Part C-Emerging Technologies* **83**, 77-103 (2017).

19. Nicolaides D, Madhusudhanan AK, Na XX, Miles J, Cebon D. Technoeconomic Analysis of Charging and Heating Options for an Electric Bus Service in London. *Ieee Transactions on Transportation Electrification* **5**, 769-781 (2019).

20. Abdelaty H, Mohamed M. A framework for BEB energy prediction using low-resolution open-source data-driven model. *Transportation Research Part D-Transport and Environment* **103**, (2022).

21. Foda A, Mohamed M, Bakr M. Dynamic Surrogate Trip-Level Energy Model for Electric Bus Transit System Optimization. *Transportation Research Record*, (2022).

22. Xylia M, Leduc S, Patrizio P, Kraxner F, Silveira S. Locating charging infrastructure for electric buses in Stockholm. *Transportation Research Part C-Emerging Technologies* **78**, 183-200 (2017).

23. He Y, Liu ZC, Song ZQ. Optimal charging scheduling and management for a fast-charging battery electric bus system. *Transportation Research Part E-Logistics and Transportation Review* **142**, (2020).

24. Liu ZC, Song ZQ, He Y. Economic Analysis of On-Route Fast Charging for Battery Electric Buses: Case Study in Utah. *Transportation Research Record* **2673**, 119-130 (2019).

25. Quarles N, Kockelman KM, Mohamed M. Costs and Benefits of Electrifying and Automating Bus Transit Fleets. *Sustainability* **12**, (2020).

26. QGIS. The Leading Open Source Desktop GIS. https://qgis.org/en/site/about/index.html. (2023)

27. Free-Software-Foundation. GNY General Public License, Version 2. 1991. https://www.gnu.org/licenses/old-licenses/gpl-2.0.en.html. (2023)

28. Town-of-Oakville. Oakville Transit Route and Schedule Information. 2019. https://portal-exploreoakville.opendata.arcgis.com/datasets/d78a1c1ad6a940009de8b68839a8f606. (2023)

29. Town-of-Oakville. Open Data License. 2014. https://www.oakville.ca/town-hall/town-studies-plans-projects/town-initiatives/open-data/open-data-licence/. (2023)

30. OpenStreetMap. Copyright and License. 2010. https://www.openstreetmap.org/copyright. (2023)

31. Foda A, Abdelaty H, Mohamed M, El-Saadany E. A generic cost-utility-emission optimization for electric bus transit infrastructure planning and charging scheduling. *Energy* **277**, (2023).

32. City-of-Guelph. Guelph Transit GTFS Data. 2016. http://data.open.guelph.ca/dataset/guelph-transit-gtfs-data. (2023)

33. City-of-Guelph. City of Guelph Open Governmen License. 2004. http://data.open.guelph.ca/pages/open-government-licence. (2023)
